# Evaluating the antibacterial effect of meropenem-loaded chitosan/sodium tripolyphosphate (TPP) nanoparticles on *Acinetobacter baumannii* isolated from hospitalized patients

**DOI:** 10.1186/s12879-024-09522-7

**Published:** 2024-06-24

**Authors:** Marziyeh Sadat Amini, Majid Baseri Salehi, Nima Bahador

**Affiliations:** 1 Department of Microbiology, Kazeroun Branch, Islamic Azad University, Kazeroun, Iran; 2 Department of Microbiology, Shiraz Branch, Islamic Azad University, Shiraz, Iran

**Keywords:** *Acinetobacter baumannii*, Antibiotic resistance, Antimicrobial therapy, Nanoparticles, Chitosan

## Abstract

**Background:**

*Acinetobacter baumannii* is a health threat due to its antibiotic resistance. Herein, antibiotic susceptibility and its association with the Toxin-antitoxin (TA) system genes in *A. baumannii* clinical isolates from Iran were investigated. Next, we prepared meropenem-loaded chitosan nanoparticles (MP-CS) and investigated their antibacterial effects against meropenem-susceptible bacterial isolates.

**Methods:**

Out of 240 clinical specimens, 60 *A. baumannii* isolates were assessed. Antibiotic resistance of the isolates against conventional antibiotics was determined alongside investigating the presence of three TA system genes (*mazEF*, *relBE*, and *higBA*). Chitosan nanoparticles were characterized in terms of size, zeta potential, encapsulation efficiency, and meropenem release activity. Their antibacterial effects were assessed using the well diffusion method, minimum inhibitory concentration (MIC), and colony-forming unit (CFU) counting. Their cytotoxic effects and biocompatibility index were determined via the MTT, LDH, and ROS formation assays.

**Results:**

Ampicillin, ceftazidime, and colistin were the least effective, and amikacin and tobramycin were the most effective antibiotics. Out of the 60 isolates, 10 (16.7%), 5 (8.3%), and 45 (75%) were multidrug-resistant (MDR), extensively drug-resistant (XDR), and pandrug-resistant (PDR), respectively. TA system genes had no significant effect on antibiotic resistance. MP-CS nanoparticles demonstrated an average size of 191.5 and zeta potential of 27.3 mV alongside a maximum encapsulation efficiency of 88.32% and release rate of 69.57%. MP-CS nanoparticles mediated similar antibacterial effects, as compared with free meropenem, against the *A. baumannii* isolates with significantly lower levels of meropenem. MP-CS nanoparticles remarkably prevented A549 and NCI-H292 cell infection by the *A. baumannii* isolates alongside demonstrating a favorable biocompatibility index.

**Conclusion:**

Antibiotic-loaded nanoparticles should be further designed and investigated to increase their antibacterial effect against *A. baumannii* and assess their safety and applicability in vivo settings.

## Introduction

*Acinetobacter baumannii* is an important nosocomial pathogen that causes various infections including pneumonia, meningitis, sepsis, wound infection, urinary tract infections (UTI), and ventilator-associated pneumonia (VAP). In the case of *A. baumannii*, increased antibiotic resistance and stability under harsh environmental conditions are considered to be associated with pathogenesis [1, 2]. Recently, the emergence of antibiotic resistance in *A. baumannii* has become a major health concern [2, 3].

Toxin-antitoxin (TA) systems are small genetic elements present on plasmids or chromosomes of various prokaryotes. In bacteria, TA systems consist of two sets of genes; one set is responsible for coding a “toxin” and the other one is responsible for the expression of a corresponding “antitoxin” [4]. TA systems function as a mechanism to immediately respond to environmental stress including antibiotics and host immune responses [5, 6]. The toxin produced in this system is stable, while the antitoxin is unstable; hence, susceptible to proteolytic degradation [4]. When the antitoxin is degraded, the produced toxin kills the bacteria [7, 8]. Although the TA systems have been reported to be associated with bacterial resistance to agents such as antibiotics, the exact underlying mechanism by which this resistance is conferred has not yet been elucidated [9, 10]. Moreover, *A. baumannii* is equipped with various TA systems existing on plasmids and/or bacterial chromosomes. These systems belong to the type II TA systems. Among the TA systems found in *A. baumannii*, three genes, namely *mazEF*, *relBE*, and *higBA*, are the most investigated and important ones that belong to the type II TA system [5, 7, 11–14]. Previous investigations have studied the relationship between these genes and antibiotic resistance and have asserted that further investigations are necessary. For instance, Coskun and colleagues reported that *Pseudomonas aeruginosa* and *Staphylococcus* isolates sensitive to gentamicin, ciprofloxacin, levofloxacin, clindamycin, phosphomycine, nitrofurantoin, fusidic acid, cefoxitin expressed relatively higher levels of the *mazEF* gene compared with those of the resistant isolates [11]. Based on the findings of a more recent report by Coşkun and colleagues, *P. aeruginosa* isolates sensitive to imipenem and meropenem expressed significantly higher levels of the *relBE* gene while isolates sensitive to amikacin significantly expressed higher levels of the *higBA* gene [13]. Regarding *A. baumannii*, Ghafourian and colleagues reported that the *mazEF* gene was expressed by 85 clinical isolates and that this TA system-related expression should be further investigated as an antibacterial therapeutic strategy [7]. However, unlike *P. aeruginosa* and *Staphylococcus*, it has not yet been elucidated if there is a correlation between the expression of the *mazEF*, *relBE*, and *higBA* genes and the resistance of *A. baumannii* isolates to common antibiotics, which was our aim in the current study.

Chitosan (CS) is a natural cationic polysaccharide with amino groups. This property gives chitosan biological activity at low pH levels allowing it to interact with negatively charged molecules including proteins, polysaccharides, and phospholipids found in bacterial cells [15–17]. The high biological activity and biocompatibility of chitosan render it a strong candidate as an antibacterial substitute for conventional therapies [15–17]. Various studies have demonstrated the applicability of chitosan nanoparticles for antibacterial purposes. For instance, Fu and colleagues have also demonstrated that chitosan-modified polymyxin B-loaded liposomes have significantly higher antibacterial effects against biofilm-forming *A. baumannii* as compared with polymyxin B alone [18]. Other researchers have also demonstrated that chitosan could be introduced as a potent inhibitor of multidrug-resistant (MDR) *A. baumannii* [15, 19]. Alongside chitosan, other nanoparticles have also been investigated for their antibacterial effects. For instance, Tiwari and colleagues developed polyvinylpyrrolidone-capped silver nanoparticles and demonstrated that they can be a potent alternative for carbapenem in combating carbapenem-resistant *A. baumannii* [20].

Based on the increasing importance of MDR *A. baumannii* in causing various infections, in this study, the prevalence of the type II TA system-related genes *mazEF*, *relBE*, and *higBA* was investigated in the clinical isolates of *A. baumannii* from patients hospitalized in Iran. In addition, the antibiotic susceptibility patterns of these isolates against various conventional antibiotics were also determined. Next, we evaluated the antibacterial effects of meropenem-loaded chitosan/sodium tripolyphosphate (TPP) nanoparticles (hereafter referred to as MP-CS nanoparticles) on a number of these clinical isolates.

## Materials and methods

### Sample collection

In the present study, specimens were collected from patients admitted to different wards of *Dey Hospital* in Tehran, Iran, from January to December 2020. The clinical specimens were obtained from patients who had been hospitalized for at least three consecutive days, and following the confirmation of *A. baumannii* infection, the bacterial isolates were given to us by the hospital’s *Infectious Disease Laboratory* for the rest of the experiments. All protocols and experiments carried out in this study were in accordance with the guidelines of the *Ethical Committee of the Islamic Azad University of Kazerun, Kazerun Branch, Kazerun, Iran* as well as approved by the mentioned committee (*approval ID: IR.IAU.KAU.REC.1399.105*).

### Bacterial isolation and characterization

For *A. baumannii* isolation, clinical specimens were separately cultured on MacConkey agar (Merck KGaA, Darmstadt, Germany) and blood agar media (Merck KGaA, Darmstadt, Germany) containing 5% sheep blood (Tamad Kala, Tehran, Iran) and incubated at 37 °C for 24 h. After the incubation, the culture plates were examined using Gram staining as *A. baumannii* are Gram-negative and they appear as short, almost round, rod-shaped Gram-negative bacterium [21]. The suspected colonies were selected for further examination which included culturing on differential culture media such as Triple Sugar Iron (TSI) agar (Merck KGaA, Darmstadt, Germany), Oxidative Fermentative (O-F) media (Merck KGaA, Darmstadt, Germany), and Sulfide Indole Motility (SIM) media (Merck KGaA, Darmstadt, Germany). Moreover, the evaluation of catalase and oxidase activity of the suspected colonies was used for further confirmation. Furthermore, determination of *Acinetobacter* species was carried out using the carbohydrate fermentation test [22]. The isolated bacteria were stored at -70 °C in Tryptic Soy Broth (TSB; Merck KGaA, Darmstadt, Germany) containing 30% glycerol until further use.

### Antibiotic susceptibility

#### Disk diffusion

The susceptibility of the *A. baumannii* isolates to antibiotics including ampicillin-sulbactam (10/10 µg), cefotaxime (30 µg), minocycline (30 µg), ceftriaxone (30 µg), ceftazidime (30 µg), meropenem (10 µg), ticarcillin-clavulanate (75/10 µg), gentamicin (10 µg), ciprofloxacin (5 µg), trimethoprim-sulfamethoxazole (1.25/23.75 µg), cefepime (30 µg), piperacillin (100 µg), amikacin (30 µg), and tobramycin (10 µg) was determined using the Kirby-Bauer disk diffusion method (DDM) on Mueller-Hinton agar (Merck KGaA, Darmstadt, Germany) according to the Clinical and Laboratory Standards Institute (CLSI) guidelines [23]. *A. baumannii* (ATCC19606) was utilized as a control during antibiotic susceptibility tests [24].

#### Minimum inhibitory concentration (MIC)

The MIC values were determined using the broth microdilution method and the clinical isolates were designated as susceptible, intermediate, or resistant according to the standards of the CLSI [23]. Briefly, ampicillin-sulbactam (MIC values of ≤ 8/4, 16/8, and ≥ 32/16 µg/mL were designated as susceptible, intermediate, and resistant, respectively), cefotaxime (MIC values of ≤ 8, 16–32, and ≥ 64 µg/mL were designated as susceptible, intermediate, and resistant, respectively), ceftriaxone (MIC values of ≤ 8, 16–32, and ≥ 64 µg/mL were designated as susceptible, intermediate, and resistant, respectively), ceftazidime (MIC values of ≤ 8, 16, and ≥ 32 µg/mL were designated as susceptible, intermediate, and resistant, respectively), meropenem (MIC values of ≤ 2, 4, and ≥ 8 µg/mL were designated as susceptible, intermediate, and resistant, respectively), ticarcillin-clavulanate (MIC values of ≤ 16/2, 32/2–64/2, and ≥ 128/2 µg/mL were designated as susceptible, intermediate, and resistant, respectively), gentamicin (MIC values of ≤ 4, 8, and ≥ 16 µg/mL were designated as susceptible, intermediate, and resistant, respectively), ciprofloxacin (MIC values of ≤ 1, 2, and ≥ 4 µg/mL were designated as susceptible, intermediate, and resistant, respectively), trimethoprim/sulfamethoxazole (MIC values of ≤ 2/38 and ≥ 4/76 µg/mL were designated as susceptible and resistant, respectively), cefepime (MIC values of ≤ 8, 16, and ≥ 32 µg/mL were designated as susceptible, intermediate, and resistant, respectively), piperacillin (MIC values of ≤ 16, 32–64, and ≥ 128 µg/mL were designated as susceptible, intermediate, and resistant, respectively), amikacin (MIC values of ≤ 16, 32, and ≥ 64 µg/mL were designated as susceptible, intermediate, and resistant, respectively), tobramycin (MIC values of ≤ 4, 8, and ≥ 16 µg/mL were designated as susceptible, intermediate, and resistant, respectively), minocycline (MIC values of ≤ 4, 8, and ≥ 16 µg/mL were designated as susceptible, intermediate, and resistant, respectively), and colistin (MIC values of ≤ 2 and ≥ 4 µg/mL were designated as intermediate and resistant, respectively) were the investigated antibiotics.

### Determination of the MDR, extensively drug-resistant (XDR), and pandrug-resistant (PDR) isolates

The MDR, XDR, and PDR status of the *A. baumannii* isolates were defined according to a previous report by Magiorakos and colleagues [25]. According to the report, “*MDR was defined as acquired non-susceptibility to at least one agent in three or more antimicrobial categories*”, while “*XDR was defined as non-susceptibility to at least one agent in all but two or fewer antimicrobial categories (i.e. bacterial isolates remain susceptible to only one or two categories)*” and “*PDR was defined as non-susceptibility to all agents in all antimicrobial categories*” [25].

### DNA extraction and PCR-assisted confirmation of the TA system genes

The bacterial isolates were cultured in TSB media overnight. DNA extraction was carried out on the fresh overnight cultures of the bacterial isolates using a commercial DNA extraction kit (CinnaGen, Tehran, Iran) according to the manufacturer’s instructions. The extracted DNA was used as the template for conventional PCR assay for determining the presence of *mazEF*, *relBE*, and *higBA* genes. The primers used for the PCR assay were previously designed by Maghraby et al. (Table 1) [26]. For each round of PCR, 3 µg of the template DNA, 2.5 µL of 10X PCR buffer, 0.5 µL of 10 mM dNTPs, 0.75 µL of 50 mM MgCl_2_, 0.25 µL of 5 U/µL of Taq DNA polymerase, and 25 pmol of each primer (forward and reverse) were mixed and brought to a final volume of 25 µL using sterile DNase/RNase-free double-distilled water (DDW). The PCR amplification assay was carried out with the following thermal conditions: an initial denaturation step at 94 °C for 1 min, followed by 30 cycles consisting of a denaturation step at 94 °C for 1 min, an annealing step at 54 °C for 1 min, and an extension step at 72 °C for 1.5 min, all of which were followed by a final extension step at 72 °C for 10 min. The amplicons were assessed by electrophoresis on 1.5% agarose gel containing 0.5 µg/mL ethidium bromide.

**Table 1:** Sequence of the primers used to screen the investigated genes

Target gene	Primer designation	Sequence (5’ to 3’)	Amplicon size (bp)	Reference
*mazEF*	mazEF-F	ACCTTCGAAGGAACTACGTCAGTAG	436	[1, 2]
	mazEF-R	ATAGGCGAACATGCAAGAAAAGGCAGC		
*relBE*	relBE-F	ATGAAGTGAACGGTCAACAATA	578	
	relBE-R	ACAGACCTCGGAAAGTGGTCG		
*higBA*	higBA-F	AGCACATCCGTACGATCTACTGC	440	
	higBA-R	TGCACTCCTGCGATGCGGCGAA		

### Nanoparticle preparation

In this experiment, the ionic gelation method was used for the preparation of the nanoparticles, as previously described by Elnaggar et al. [27]. In detail, 1 g of chitosan (Sigma Aldrich, United States) was dissolved in 200 mL of acetic acid solution (2%) and was stirred for 30 min at room temperature. Next, 0.2% w/v sodium tripolyphosphate (TPP) solution (prepared using deionized water) was added to the chitosan solution under stirring in a drop-wise manner. The resultant thick emulsion of the prepared chitosan/TPP nanoparticles was allowed to sediment which was followed by ultracentrifugation (Beckman, California, US) at 25,000 rpm for 30 min to purify the nanoparticles. The precipitate of the purified nanoparticles was stored at 4 ˚C until further use.

For the preparation of the meropenem-loaded chitosan/TPP nanoparticles, a 0.015% w/v chitosan solution was prepared using 2% v/v acetic acid. Next, 0.006% w/v sodium TPP solution was prepared using deionized water, and different concentrations of meropenem (with final concentrations of 0.5, 1, and 1.85 µg) were added to it under continuous stirring [28]. The resultant sodium TPP/meropenem solution was added to the chitosan solution under stirring for 30 min in a drop-wise manner. The resultant thick emulsion of the prepared meropenem-loaded chitosan/TPP nanoparticles was allowed to sediment and was centrifuged at 25,000 rpm for 30 min to purify the nanoparticles. The precipitate of the purified nanoparticles was stored at 4 ˚C until further use.

### Characterization of nanoparticles

#### Particle size, polydispersity index (PDI), and ζ potential

The particle size, PDI, and ζ potential of the prepared nanoparticles were determined by the dynamic light scattering technique using a particle analyzer. Of note, the nanoparticle-containing samples were appropriately diluted with double distilled water before the measurements, and all of the measurements were performed in triplicate.

#### Encapsulation efficiency

A previously described method was used to evaluate the encapsulation efficiency of the prepared nanoparticles [29, 30]. First, the nanoparticles were diluted with phosphate-buffered saline (PBS; 1:10 v/v). Next, the samples were centrifuged at 15,000 rpm at 4 ˚C for 15 min. The absorption of the resultant supernatant was measured by a UV-visible spectrophotometer instrument (UV-1800, Shimadzu, Japan) at the wavelength of 260 nm to measure the unentrapped drug. Of note, methanol was used as the blank. Finally, the encapsulation efficiency of the prepared nanoparticles was calculated using the following formula:

$${\text{Encapsulation efficiency}}\,(\% )\, = \,\frac{{total\,unentrapped\,drug}}{{toral\,drug}} \times 100$$

#### In vitro drug release

The release rate of meropenem from the prepared meropenem-loaded chitosan/TPP nanoparticles was measured using the membrane diffusion method as previously described [30]. The absorbance of each of the samples was measured at least three times at the wavelength of 298 nm to calculate the concentration of meropenem [31].

### Antibacterial effects of the nanoparticles

The in vitro antibacterial effects of the prepared nanoparticles were assessed using the well diffusion method as previously described [32, 33]. The Mueller Hinton agar plates were prepared. Following the solidification of the prepared plates, colonies of *A. baumannii* isolates were selected from overnight culture plates and inoculated on Mueller Hinton agar plates. Next, wells were punched out using a 0.7 cm cork borer. 100 µL of each nanoparticle sample, or meropenem (at the final concentration of 10 µg as per the CLSI guidelines), were separately pipetted into different wells [23]. Finally, the plates were incubated at 37 °C for 12 h. After this, the radius of the clear bacterial inhibition zone around each of the wells was carefully measured. Furthermore, the MIC values against the bacterial isolates were evaluated using the microdilution method to further determine the antibacterial effects of the prepared nanoparticles. Of note, in both well diffusion and microdilution tests, meropenem and double-distilled water were used as the positive and negative control, respectively.

### Cell culture

The A549 cell line (which is a human alveolar basal epithelial cell line) and the NCI-H292 cell line (which is a mucoepidermoid pulmonary carcinoma cell line) were obtained from the *Iranian Biological Resource Center* (*IBRC*; Tehran, Iran) and *American Type Culture Collection* (*ATCC*, United States), respectively. These two cell lines were selected as they have been previously used as cell models for *A. baumannii* infection [34–38]. The A549 cells were cultured in high glucose Dulbecco’s Modified Eagle Medium (DMEM; Gibco, United States) which was supplemented with 10% (v/v) heat-inactivated fetal bovine serum (FBS; Gibco, California, United States). Moreover, the NCI-H292 cells were cultured in RPMI-1640 (Gibco, United States) supplemented with 10% FBS. Additionally, the culture media was supplemented with amphotericin B (2.5 µg/mL), vancomycin (50 µg/mL), gentamicin (50 µg/mL), and 1% 4-(2-hydroxyethyl)piperazine-1-ethane-sulfonic acid (HEPES). All of the cell cultures were incubated in an incubator at 37 ˚C with 5% CO_2_ in a humidified condition.

### Cell line and bacterial culture preparation for infection

A549 and NCI-H292 cells in the growth phase were washed twice using PBS. Next, the cells were detached using a 0.05% trypsin-ethylenediaminetetraacetic acid (EDTA) solution and were centrifuged at 1500 rpm for 5 minutes. The resultant cell pellet was resuspended in room temperature DMEM (supplemented with 10% v/v FBS but without antibiotics) to obtain a cell concentration of 2 × 10^5^ cells/mL. At the same time, the bacterial isolates were grown in 100 mL of LB broth at 37 ˚C (with 200 rpm agitation) until an optical density of 0.6 at the wavelength of 600 nm was obtained. The colony-forming unit (CFU) of the bacterial isolates was calculated before the infection experiment. Of note, *A. baumannii* ATCC 19,606 was used as the reference bacteria. Moreover, from the clinical isolates susceptible to meropenem, two isolates were selected for the further steps of the investigations.

### Cell infection

A549 and NCI-H292 cells were cultured until reaching a contamination-free confluency rate of more than 90%. Next, the cells were washed twice with PBS, and 1 mL of fresh room temperature DMEM (supplemented with 10% v/v FBS but without antibiotics) was added to each well (6-well cell culture plates). The bacterial isolates were added to the cell-containing wells with a multiplicity of infection (MOI) of 3:1 indicating 1.2 × 10^7^ bacteria CFUs:0.4 × 10^7^ A549 cells. The plates were incubated at 37 ˚C containing 5% CO_2_ for 24 h.

### CFU counting

The population of the bacterial isolates after infection was classified into four categories which include [1] before coinfection [2], remaining non-interacting during coinfection [3], interacting or adhered bacteria on the surface of A549 or NCI-H292 cells, and [4] bacteria internalized into the cells. Of note, there were four different methods used for the calculation of CFU of the bacteria of each of these four categories. Briefly, for the CFU calculation of bacteria remaining non-interacting during coinfection, the suspension culture in DMEM media which contained the free bacteria was used while for the CFU calculation of interacting or adhered bacteria on the surface of the cells, the cell culture media were gently first aspirated and the cells were detached and centrifuged. Next, the cell pellet was resuspended again and a sample of the solution was used for CFU calculation. Ultimately, for the CFU calculation of bacteria internalized into the cells, samples were taken after the homogenization of the cells. Of note, CFU calculation was performed according to a method previously reported by Tiwari and colleagues [20].

### Cytotoxic effect of the nanoparticles on A549 and NCI-H292 cells

The in vitro cytotoxicity of the MP-CS nanoparticles on the A549 and NCI-H292 cell lines was investigated using the MTT assay (MTT Assay Kit; Abcam, Cambridge, UK) and LDH assay (LDH Assay Kit; Abcam, Cambridge, UK) as per the manufacturer’s instructions. Briefly, the effects of the different concentrations of the nanoparticles on the viability of the A549 and NCI-H292 cells were investigated to determine the IC50 and the non-toxic dose of the MP-CS nanoparticles. Of note, the cells were seeded in 96-well cell culture plates for 24 h for both of the assays.

### **Evaluation of reactive oxygen species** (**ROS) formation**

The cells were seeded in the wells of an ELISA plate and after adhering to the bottom of the wells, a proportion of the culture medium was replaced with 50 µL of the 0.5% nitroblue tetrazolium (NBT) solution. The plate was incubated for 1.5 h and then the supernatant was carefully discarded. Next, the wells were supplied with 200 µL absolute methanol for cell fixation and then washed twice with PBS. The wells were left to dry at room temperature, after which the cells were exposed to 150 µL KOH (2 M) for cell membrane disruption. In the following, 150 µL dimethyl sulfoxide (DMSO; Sigma-Aldrich, United States) was added to each well for the dissolution of the formazan crystals. Ultimately, the absorbance of each well was measured at 620 nm.

### Statistical analysis

The data pertaining to the association between the risk factors were analyzed by Chi-square with a confidence level of 95% using the IBM SPSS statistics software (version 27; IBM, United States). Moreover, one-way ANOVA and Student’s t-test were used for statistical analysis between the experimental groups as performed using the GraphPad Prism software (version 10.1.0; GraphPad Software, San Diego, California, United States). A p-value < 0.05 was considered statistically significant.

## Results

### Demographic data

A total number of 240 clinical specimens (of which 88, 53, 45, 33, and 21 specimens were derived from the blood, urine, sputum, respiratory secretions, and skin wounds of the patients, respectively) were collected from 60 patients; out of which, a total number of 60 *A. baumannii* isolates were selected randomly in a way that no more than one isolate was selected from each patient. Out of the selected *A. baumannii* isolates, 24 belonged to female patients (40%) and 36 belonged to male patients (60%).

Among them, the most bacterial isolates were derived from trachea (31; 51.7%), throat (6; 10%), sputum (5; 8.3%), urinary catheterization (5; 8.3%), axillary (3; 5%), abdominal wound (2; 3.3%), chest wound (2; 3.3%), inguinal (2; 3.3%), central venous port (1; 1.7%), nose (1; 1.7%), nasogastric tube (1; 1.7%), and sternum (1; 1.7%). Among the causes of hospitalization (Table 2), respiratory issues (17; 28.3), heart surgery (6; 10%), heart issues (6; 10%), abdominal surgery (6; 10%), cancer (6; 10%), and stroke (5; 8.3%) were the most frequent causes of hospitalization of the patients infected with *A. baumannii.*

**Table 2:** The sex and cause of hospitalization of the patients infected with *A. baumannii*

Cause of hospitalization	Female patients (%)	Male patients (%)	Total (%)
Abdominal surgery	2 (3.3%)	4 (6.7%)	6 (10%)
Aortic valve surgery	1 (1.7%)	0 (0%)	1 (1.7%)
Brain issues	1 (1.7%)	0 (0%)	1 (1.7%)
Brain surgery	0 (0%)	1 (1.7%)	1 (1.7%)
Cancer	2 (3.3%)	4 (6.7%)	6 (10%)
Diabetes	1 (1.7%)	1 (1.7%)	2 (3.3%)
Heart issues	1 (1.7%)	5 (8.3%)	6 (10%)
Stroke	1 (1.7%)	4 (6.7%)	5 (8.3%)
Heart surgery	3 (5%)	3 (5%)	6 (10%)
Orthopedic surgery	0 (0%)	1 (1.7%)	1 (1.7%)
Parturition	1 (1.7%)	0 (0%)	1 (1.7%)
Pelvic surgery	1 (1.7%)	1 (1.7%)	2 (3.3%)
Premature infant	1 (1.7%)	1 (1.7%)	2 (3.3%)
Prostate castration	0 (0%)	1 (1.7%)	1 (1.7%)
Respiratory issues	7 (11.7%)	10 (16.7%)	17 (28.3%)
Plastic surgery	2 (3.3%)	0 (0%)	2 (3.3%)
Total	24 (40%)	36 (60%)	60 (100%)

### Antibiotic resistance patterns

According to the results (Tables 3 and 4), ampicillin-sulbactam (60; 100%), colistin (60; 100%), ceftazidime (60; 100%), cefotaxime (59; 98.3%), ceftriaxone (59; 98.3%), piperacillin (59; 98.3%), ticarcillin-clavulanate (59; 98.3%), minocycline (59; 98.3%), gentamicin (58; 96.7%), cefepime (58; 96.7%), meropenem (58; 96.7%), and ciprofloxacin (58; 96.7%) were among the used antibiotics to which most of the *A. baumannii* isolates showed resistance. Moreover, amikacin (51; 85%) and tobramycin (51; 85%) were the tested antibiotics against which the lowest rates of resistance were observed. In this study, out of the 60 bacterial isolates, 59 isolates (98.34%) were resistant to more than eight of the tested antibiotics and only 1 isolate (1.66%) demonstrated susceptibility to seven of the tested antibiotics.

**Table 3:** The MIC values of the investigated antibiotics against the *A. baumannii* clinical isolates. All values are presented in µg/mL alongside their antibiotic resistance status according to the CLSI standards (R and S represent resistant and susceptible isolates, respectively)

Antimicrobial category	Aminoglycosides			Cephalosporins				Carbapenems	Penicillins			Fluoroquinolones	Sulfonamide	Tetracyclines	Polymyxins
Antimicrobial agent	Gentamicin	Amikacin	Tobramycin	Cefotaxime	Ceftriaxone	Ceftazidime	Cefepime	Meropenem	Ampicillin-sulbactam	Piperacillin	Ticarcillin-clavulanate	Ciprofloxacin	Trimethoprim/sulfamethoxazole	Minocycline	Colistin
Isolate 1	22.31 (R)	69.05 (R)	29.12 (R)	73.26 (R)	79.70 (R)	38.36 (R)	41.64 (R)	9.29 (R)	41.01/17.90 (R)	147.31 (R)	140.82/3.72 (R)	4.67 (R)	5.00/82.27 (R)	17.23 (R)	4.94 (R)
Isolate 2	3.29 (S)	12.99 (S)	2.19 (S)	72.48 (R)	67.06 (R)	44.73 (R)	4.16 (S)	12.59 (R)	41.07/17.32 (R)	135.05 (R)	132.49/3.66 (R)	0.74 (S)	4.76/90.75 (R)	16.35 (R)	4.78 (R)
Isolate 3	30.87 (R)	70.25 (R)	25.62 (R)	78.33 (R)	70.40 (R)	43.10 (R)	39.39 (R)	9.41 (R)	38.33/17.60 (R)	156.59 (R)	132.36/3.80 (R)	4.61 (R)	6.73/92.40 (R)	18.49 (R)	4.37 (R)
Isolate 4	19.31 (R)	69.82 (R)	20.23 (R)	68.94 (R)	74.42 (R)	34.51 (R)	41.92 (R)	12.02 (R)	38.85/21.73 (R)	133.35 (R)	139.11/2.91 (R)	6.92 (R)	4.83/87.09 (R)	18.89 (R)	5.91 (R)
Isolate 5	30.04 (R)	80.12 (R)	21.10 (R)	75.33 (R)	81.13 (R)	46.19 (R)	42.58 (R)	12.17 (R)	38.56/23.26 (R)	154.45 (R)	137.99/3.13 (R)	5.87 (R)	5.36/78.12 (R)	19.51 (R)	6.37 (R)
Isolate 6	27.90 (R)	10.31 (S)	3.41 (S)	6.16 (S)	69.13 (R)	36.17 (R)	38.64 (R)	8.74 (R)	36.24/19.84 (R)	141.94 (R)	13.32/1.49 (S)	4.49 (R)	4.89/93.65 (R)	16.99 (R)	4.72 (R)
Isolate 7	22.85 (R)	77.66 (R)	20.72 (R)	70.83 (R)	73.16 (R)	36.16 (R)	40.74 (R)	10.57 (R)	41.18/17.56 (R)	140.39 (R)	141.61/2.70 (R)	6.98 (R)	6.33/81.59 (R)	19.01 (R)	5.07 (R)
Isolate 8	19.27 (R)	71.79 (R)	23.33 (R)	71.92 (R)	68.29 (R)	40.64 (R)	38.00 (R)	9.23 (R)	39.91/21.15 (R)	142.94 (R)	132.67/2.98 (R)	5.52 (R)	4.66/88.17 (R)	16.68 (R)	6.17 (R)
Isolate 9	25.78 (R)	81.55 (R)	19.51 (R)	72.75 (R)	72.95 (R)	44.68 (R)	37.95 (R)	10.07 (R)	39.70/19.72 (R)	136.25 (R)	143.82/2.32 (R)	4.45 (R)	6.05/93.36 (R)	18.76 (R)	4.64 (R)
Isolate 10	27.21 (R)	77.29 (R)	26.48 (R)	76.47 (R)	80.66 (R)	35.63 (R)	41.10 (R)	8.58 (R)	39.83/23.33 (R)	156.91 (R)	136.10/2.74 (R)	6.93 (R)	6.20/78.20 (R)	19.86 (R)	5.29 (R)
Isolate 11	30.02 (R)	69.29 (R)	23.14 (R)	78.91 (R)	68.67 (R)	37.95 (R)	36.36 (R)	11.72 (R)	39.53/19.96 (R)	134.04 (R)	131.74/2.85 (R)	6.43 (R)	7.49/79.59 (R)	18.93 (R)	6.29 (R)
Isolate 12	26.64 (R)	70.78 (R)	25.97 (R)	71.24 (R)	73.64 (R)	35.88 (R)	41.84 (R)	11.65 (R)	35.71/17.92 (R)	141.68 (R)	144.29/3.51 (R)	6.13 (R)	6.46/90.61 (R)	16.59 (R)	5.21 (R)
Isolate 13	21.51 (R)	74.48 (R)	17.63 (R)	71.31 (R)	68.91 (R)	39.60 (R)	42.76 (R)	9.26 (R)	40.33/22.61 (R)	152.32 (R)	137.33/4.01 (R)	6.34 (R)	7.08/83.05 (R)	17.79 (R)	4.99 (R)
Isolate 14	25.74 (R)	66.95 (R)	26.24 (R)	74.34 (R)	80.02 (R)	47.82 (R)	43.21 (R)	8.79 (R)	41.41/22.66 (R)	153.26 (R)	142.66/3.48 (R)	5.88 (R)	5.69/91.35 (R)	18.01 (R)	5.92 (R)
Isolate 15	23.90 (R)	67.73 (R)	21.06 (R)	67.76 (R)	74.18 (R)	35.16 (R)	41.28 (R)	10.99 (R)	38.25/19.35 (R)	145.50 (R)	140.50/3.06 (R)	7.07 (R)	6.02/80.24 (R)	19.83 (R)	5.57 (R)
Isolate 16	27.38 (R)	66.92 (R)	22.81 (R)	77.04 (R)	77.25 (R)	35.57 (R)	38.52 (R)	12.44 (R)	41.46/22.14 (R)	151.48 (R)	140.71/3.08 (R)	6.67 (R)	5.62/84.25 (R)	16.74 (R)	6.24 (R)
Isolate 17	28.90 (R)	68.30 (R)	19.26 (R)	77.84 (R)	70.17 (R)	42.88 (R)	38.89 (R)	9.85 (R)	39.94/18.13 (R)	143.44 (R)	131.65/4.09 (R)	4.51 (R)	5.59/92.50 (R)	18.92 (R)	4.80 (R)
Isolate 18	21.39 (R)	78.03 (R)	27.97 (R)	75.71 (R)	76.13 (R)	39.89 (R)	39.47 (R)	11.81 (R)	37.22/21.24 (R)	152.93 (R)	131.83/2.48 (R)	4.37 (R)	6.59/88.11 (R)	19.14 (R)	6.04 (R)
Isolate 19	22.64 (R)	74.21 (R)	20.05 (R)	79.14 (R)	72.70 (R)	43.41 (R)	43.23 (R)	11.23 (R)	35.80/23.08 (R)	156.20 (R)	143.10/2.54 (R)	6.39 (R)	4.58/79.21 (R)	19.28 (R)	4.91 (R)
Isolate 20	21.83 (R)	70.55 (R)	28.21 (R)	67.81 (R)	80.26 (R)	46.60 (R)	40.59 (R)	10.11 (R)	41.48/22.90 (R)	135.53 (R)	133.37/2.40 (R)	5.89 (R)	6.36/94.04 (R)	18.06 (R)	4.15 (R)
Isolate 21	28.50 (R)	66.80 (R)	26.21 (R)	69.01 (R)	74.35 (R)	38.29 (R)	34.68 (R)	11.59 (R)	35.97/22.86 (R)	158.57 (R)	140.80/4.19 (R)	4.77 (R)	6.95/84.66 (R)	19.13 (R)	4.20 (R)
Isolate 22	25.94 (R)	68.91 (R)	28.70 (R)	79.02 (R)	74.20 (R)	42.84 (R)	42.10 (R)	11.30 (R)	36.94/18.59 (R)	152.37 (R)	133.74/3.67 (R)	6.08 (R)	5.49/80.32 (R)	19.25 (R)	5.90 (R)
Isolate 23	20.83 (R)	66.94 (R)	22.39 (R)	73.31 (R)	79.30 (R)	37.28 (R)	34.58 (R)	10.79 (R)	39.13/19.76 (R)	138.30 (R)	144.17/2.90 (R)	7.05 (R)	4.65/93.10 (R)	19.02 (R)	5.85 (R)
Isolate 24	18.91 (R)	67.36 (R)	20.67 (R)	69.23 (R)	71.23 (R)	37.81 (R)	39.65 (R)	12.77 (R)	40.64/22.71 (R)	138.73 (R)	136.20/3.07 (R)	4.39 (R)	5.72/78.18 (R)	19.29 (R)	4.52 (R)
Isolate 25	20.63 (R)	78.88 (R)	19.58 (R)	79.01 (R)	79.59 (R)	43.50 (R)	37.71 (R)	9.79 (R)	35.07/20.12 (R)	144.66 (R)	140.91/3.22 (R)	5.17 (R)	4.56/89.09 (R)	17.08 (R)	6.38 (R)
Isolate 26	21.16 (R)	81.07 (R)	19.16 (R)	71.95 (R)	68.13 (R)	38.30 (R)	35.16 (R)	10.52 (R)	36.50/21.95 (R)	158.68 (R)	132.02/3.81 (R)	6.79 (R)	6.62/91.86 (R)	17.25 (R)	5.50 (R)
Isolate 27	20.96 (R)	10.85 (S)	2.86 (S)	75.97 (R)	77.68 (R)	35.60 (R)	42.94 (R)	12.35 (R)	39.68/18.67 (R)	148.93 (R)	145.64/3.71 (R)	4.62 (R)	6.18/82.74 (R)	19.56 (R)	5.09 (R)
Isolate 28	21.46 (R)	9.53 (S)	3.09 (S)	71.91 (R)	74.00 (R)	41.75 (R)	38.85 (R)	11.52 (R)	37.84/17.41 (R)	152.73 (R)	142.65/3.21 (R)	5.03 (R)	5.25/93.71 (R)	19.55 (R)	5.54 (R)
Isolate 29	20.10 (R)	66.75 (R)	20.27 (R)	75.49 (R)	76.01 (R)	41.84 (R)	43.13 (R)	11.43 (R)	38.54/23.35 (R)	142.49 (R)	141.37/4.27 (R)	4.50 (R)	5.74/80.50 (R)	19.22 (R)	5.67 (R)
Isolate 30	23.05 (R)	70.29 (R)	21.99 (R)	71.58 (R)	77.86 (R)	34.99 (R)	35.86 (R)	10.85 (R)	37.72/21.85 (R)	137.85 (R)	139.54/3.76 (R)	6.96 (R)	4.44/82.20 (R)	19.42 (R)	4.54 (R)
Isolate 31	28.23 (R)	82.02 (R)	24.95 (R)	73.79 (R)	71.25 (R)	43.69 (R)	36.39 (R)	8.33 (R)	36.31/19.50 (R)	132.00 (R)	136.29/2.82 (R)	6.10 (R)	5.31/88.54 (R)	17.64 (R)	4.68 (R)
Isolate 32	24.05 (R)	10.29 (S)	23.71 (R)	71.51 (R)	78.87 (R)	44.77 (R)	36.85 (R)	8.34 (R)	38.41/21.68 (R)	143.41 (R)	139.89/2.86 (R)	6.63 (R)	4.95/82.43 (R)	17.03 (R)	5.13 (R)
Isolate 33	27.48 (R)	11.54 (S)	1.91 (S)	74.51 (R)	78.73 (R)	38.41 (R)	43.20 (R)	11.03 (R)	36.32/22.33 (R)	133.30 (R)	143.65/3.16 (R)	6.20 (R)	4.86/81.94 (R)	19.39 (R)	6.14 (R)
Isolate 34	28.38 (R)	70.90 (R)	20.95 (R)	72.31 (R)	67.39 (R)	40.74 (R)	41.11 (R)	10.27 (R)	37.32/19.13 (R)	155.58 (R)	142.34/4.24 (R)	5.82 (R)	4.70/87.21 (R)	18.81 (R)	6.20 (R)
Isolate 35	27.87 (R)	67.86 (R)	18.57 (R)	72.70 (R)	67.63 (R)	44.71 (R)	40.76 (R)	12.38 (R)	36.30/23.27 (R)	151.24 (R)	135.13/3.28 (R)	6.53 (R)	6.92/86.07 (R)	16.36 (R)	6.33 (R)
Isolate 36	21.81 (R)	79.60 (R)	19.68 (R)	71.50 (R)	78.40 (R)	38.61 (R)	42.46 (R)	8.63 (R)	39.27/18.87 (R)	139.31 (R)	138.54/3.20 (R)	6.77 (R)	7.10/87.28 (R)	16.33 (R)	4.16 (R)
Isolate 37	31.37 (R)	70.33 (R)	19.00 (R)	76.23 (R)	71.68 (R)	47.49 (R)	34.62 (R)	8.49 (R)	40.86/23.21 (R)	136.10 (R)	140.08/2.72 (R)	5.51 (R)	6.56/82.21 (R)	18.87 (R)	5.40 (R)
Isolate 38	29.94 (R)	69.22 (R)	21.40 (R)	74.95 (R)	80.14 (R)	38.85 (R)	40.88 (R)	10.90 (R)	38.94/19.41 (R)	137.13 (R)	142.04/4.00 (R)	5.37 (R)	7.01/77.71 (R)	17.29 (R)	4.28 (R)
Isolate 39	30.70 (R)	72.19 (R)	27.80 (R)	68.86 (R)	70.76 (R)	39.38 (R)	42.80 (R)	10.43 (R)	38.88/22.24 (R)	139.03 (R)	134.63/3.88 (R)	5.38 (R)	7.11/91.92 (R)	18.59 (R)	4.12 (R)
Isolate 40	21.67 (R)	72.12 (R)	23.66 (R)	74.45 (R)	66.86 (R)	37.27 (R)	38.82 (R)	12.39 (R)	36.73/22.63 (R)	138.07 (R)	144.16/2.57 (R)	4.70 (R)	4.30/93.79 (R)	19.35 (R)	5.43 (R)
Isolate 41	31.80 (R)	12.33 (S)	2.29 (S)	76.22 (R)	76.18 (R)	47.64 (R)	43.27 (R)	8.57 (R)	39.78/19.20 (R)	136.89 (R)	143.05/2.29 (R)	5.77 (R)	6.74/90.89 (R)	18.98 (R)	6.03 (R)
Isolate 42	26.23 (R)	76.73 (R)	27.31 (R)	77.67 (R)	66.97 (R)	38.50 (R)	41.25 (R)	9.00 (R)	35.20/20.00 (R)	151.55 (R)	131.71/3.45 (R)	6.36 (R)	5.52/92.23 (R)	19.65 (R)	5.20 (R)
Isolate 43	31.51 (R)	81.86 (R)	19.30 (R)	68.25 (R)	79.25 (R)	35.43 (R)	35.96 (R)	11.46 (R)	37.33/20.71 (R)	133.03 (R)	137.11/2.65 (R)	4.58 (R)	6.68/89.56 (R)	18.64 (R)	4.71 (R)
Isolate 44	20.25 (R)	68.25 (R)	24.41 (R)	77.88 (R)	80.24 (R)	34.70 (R)	42.05 (R)	9.12 (R)	41.23/19.31 (R)	140.47 (R)	138.80/4.07 (R)	4.81 (R)	6.30/82.50 (R)	19.34 (R)	5.23 (R)
Isolate 45	29.89 (R)	67.30 (R)	3.31 (S)	69.37 (R)	81.01 (R)	45.16 (R)	40.50 (R)	12.50 (R)	38.50/20.13 (R)	149.31 (R)	137.72/3.10 (R)	6.76 (R)	5.80/90.43 (R)	17.62 (R)	5.86 (R)
Isolate 46	22.97 (R)	75.11 (R)	2.32 (S)	75.82 (R)	76.25 (R)	47.20 (R)	38.47 (R)	10.44 (R)	34.60/18.31 (R)	133.20 (R)	142.78/4.15 (R)	5.66 (R)	4.38/79.81 (R)	19.15 (R)	4.44 (R)
Isolate 47	23.04 (R)	68.66 (R)	24.25 (R)	76.12 (R)	74.06 (R)	36.96 (R)	38.84 (R)	10.37 (R)	37.12/18.33 (R)	136.37 (R)	143.73/2.97 (R)	6.18 (R)	7.24/81.64 (R)	17.95 (R)	5.24 (R)
Isolate 48	28.40 (R)	81.95 (R)	26.17 (R)	75.60 (R)	80.54 (R)	46.86 (R)	43.08 (R)	12.80 (R)	41.16/18.52 (R)	147.36 (R)	137.38/3.19 (R)	6.02 (R)	6.00/92.63 (R)	19.68 (R)	4.35 (R)
Isolate 49	30.72 (R)	71.44 (R)	26.44 (R)	67.99 (R)	68.86 (R)	44.43 (R)	42.91 (R)	12.60 (R)	36.41/20.39 (R)	136.97 (R)	132.23/3.40 (R)	4.99 (R)	5.81/89.99 (R)	17.16 (R)	4.92 (R)
Isolate 50	19.59 (R)	69.47 (R)	19.03 (R)	78.57 (R)	81.11 (R)	41.65 (R)	35.75 (R)	12.89 (R)	40.78/22.62 (R)	144.80 (R)	144.85/2.34 (R)	6.91 (R)	6.83/86.00 (R)	19.62 (R)	4.11 (R)
Isolate 51	18.92 (R)	14.29 (S)	24.78 (R)	75.26 (R)	70.00 (R)	39.67 (R)	39.08 (R)	10.55 (R)	38.07/19.05 (R)	139.42 (R)	132.19/3.30 (R)	6.23 (R)	6.85/89.73 (R)	16.34 (R)	4.67 (R)
Isolate 52	30.10 (R)	68.57 (R)	18.19 (R)	72.60 (R)	72.49 (R)	47.37 (R)	39.99 (R)	11.02 (R)	39.95/17.76 (R)	140.98 (R)	136.99/3.55 (R)	6.15 (R)	4.94/82.52 (R)	18.71 (R)	5.15 (R)
Isolate 53	22.04 (R)	73.76 (R)	19.96 (R)	77.05 (R)	68.03 (R)	46.38 (R)	38.90 (R)	9.25 (R)	36.91/22.44 (R)	149.63 (R)	132.16/3.79 (R)	7.04 (R)	4.47/77.04 (R)	16.57 (R)	4.59 (R)
Isolate 54	2.66 (S)	13.90 (S)	3.21 (S)	67.98 (R)	78.06 (R)	36.34 (R)	34.35 (R)	12.04 (R)	35.44/19.69 (R)	139.30 (R)	133.91/2.53 (R)	6.78 (R)	6.09/90.63 (R)	17.26 (R)	5.39 (R)
Isolate 55	20.82 (R)	76.54 (R)	20.50 (R)	70.01 (R)	73.74 (R)	38.00 (R)	34.91 (R)	9.28 (R)	35.54/21.46 (R)	147.52 (R)	135.57/2.92 (R)	5.76 (R)	1.18/33.12 (S)	18.70 (R)	5.97 (R)
Isolate 56	27.45 (R)	74.28 (R)	22.09 (R)	73.13 (R)	5.31 (S)	40.68 (R)	6.32 (S)	1.39 (S)	39.62/21.63 (R)	14.31 (S)	138.98/3.46 (R)	0.85 (S)	1.51/30.29 (S)	3.22 (S)	5.58 (R)
Isolate 57	29.22 (R)	81.43 (R)	22.23 (R)	76.69 (R)	69.41 (R)	47.09 (R)	41.67 (R)	1.55 (S)	35.29/22.59 (R)	152.70 (R)	136.21/2.89 (R)	7.10 (R)	1.39/18.53 (S)	18.12 (R)	5.81 (R)
Isolate 58	23.77 (R)	77.92 (R)	27.06 (R)	71.33 (R)	73.82 (R)	34.82 (R)	42.13 (R)	12.52 (R)	38.17/21.22 (R)	154.62 (R)	133.77/2.80 (R)	4.47 (R)	1.78/23.50 (S)	19.75 (R)	4.34 (R)
Isolate 59	29.55 (R)	73.33 (R)	29.40 (R)	70.68 (R)	76.33 (R)	39.21 (R)	39.91 (R)	8.75 (R)	37.83/18.42 (R)	136.09 (R)	145.38/3.03 (R)	7.01 (R)	5.37/88.93 (R)	16.79 (R)	4.47 (R)
Isolate 60	21.17 (R)	69.79 (R)	25.85 (R)	73.30 (R)	80.49 (R)	43.85 (R)	34.77 (R)	8.44 (R)	35.88/20.66 (R)	140.87 (R)	132.99/3.09 (R)	5.67 (R)	6.57/94.00 (R)	17.39 (R)	5.42 (R)

**Table 4:** The Antibiotic resistance pattern of the *A. baumannii* clinical isolates to the investigated antibiotics

Antimicrobial category	Antimicrobial agent	Count (%)			
		Resistant	Intermediate	Susceptible	Total
Aminoglycosides	Gentamicin	58 (96.7%)	0 (0%)	2 (3.3%)	60 (100%)
	Amikacin	51 (85%)	0 (0%)	9 (15%)	60 (100%)
	Tobramycin	51 (85%)	0 (0%)	9 (15%)	60 (100%)
Cephalosporins	Cefotaxime	59 (98.3%)	0 (0%)	1 (1.7%)	60 (100%)
	Ceftriaxone	59 (98.3%)	0 (0%)	1 (1.7%)	60 (100%)
	Ceftazidime	60 (100%)	0 (0%)	0 (0%)	60 (100%)
	Cefepime	58 (96.7%)	0 (0%)	2 (3.3%)	60 (100%)
Carbapenems	Meropenem	58 (96.7%)	0 (0%)	2 (3.3%)	60 (100%)
Penicillin	Ampicillin-sulbactam	60 (100%)	0 (0%)	0 (0%)	60 (100%)
	Piperacillin	59 (98.3%)	0 (0%)	1 (1.7%)	60 (100%)
	Ticarcillin-clavulanate	59 (98.3%)	0 (0%)	1 (1.7%)	60 (100%)
Fluoroquinolones	Ciprofloxacin	58 (96.7%)	0 (0%)	2 (3.3%)	60 (100%)
Sulfonamide	Trimethoprim/sulfamethoxazole	56 (93.3%)	0 (0%)	4 (6.7%)	60 (100%)
Tetracyclines	Minocycline	59 (98.3%)	0 (0%)	1 (1.7%)	60 (100%)
Polymyxins	Colistin	60 (100%)	0 (0%)	0 (0%)	60 (100%)

### Determination of the MDR, XDR, and PDR isolates

Our findings demonstrated that out of the 60 clinical isolates, 10 (16.7%), 5 (8.3%%), and 45 (75%) were MDR, XDR, and PDR, respectively. Further analyses indicated no significant relationship between the age of the patients, the sex of the patients, the hospitalization ward, and the isolation site with the MDR, XDR, and PDR status of the bacterial isolates (*p* = 0.666, *p* = 0.361, *p* = 0.606, *p* = 0.208, respectively). However, the only significant relationship between the antibiotic resistance status of the bacterial isolates was found with the hospitalization cause of the patients from whom the isolates were derived (*p* = 0.025).

### Distribution of the TA system genes

In the present study, the prevalence of the *mazEF*, *relBE*, and *higBA* genes in 60 *A. baumannii* clinical isolates was investigated. According to the results, *mazEF*, *relBE*, and *higBA* were present in 24 (40%), 32 (53.3%), and 35 (58.3%) of the bacterial isolates, respectively. Moreover, 6 (10%) clinical isolates were negative for all of the investigated genes, meaning that 54 (90%) of the clinical isolates contained at least one of the studied genes.

Further analyses indicated that there is no significant relationship between the presence of the *mazEF*, *relBE* and *higBA* genes in the clinical isolates with their resistance to the investigated antibiotic agents (Table 5). Moreover, it was demonstrated that the presence or absence of the *mazEF*, *relBE*, and *higBA* genes had no significant effect on the MDR, XDR, or PDR status of the clinical isolates (Table 6).

**Table 5:** The *mazEF, relBE*, and *higBA* prevalence of the clinical isolates and their relationship with antibiotic resistance

Antibiotic class (antibiotic agent)			Negative	Positive	Total	*P* value
	**mazEF**					
Aminoglycosides (Gentamicin)	I	Count	0	0	0	0.240219
		% of total	0%	0%	0%	
	R	Count	34	24	58	
		% of total	56.7%	40.0%	96.7%	
	S	Count	2	0	2	
		% of total	3.3%	0.0%	3.3%	
Total		Count	36	24	60	
		% of total	60.0%	40.0%	100.0%	
Aminoglycosides (Amikacin)	I	Count	0	0	0	0.657905
		% of total	0%	0%	0%	
	R	Count	30	21	51	
		% of total	50.0%	35.0%	85.0%	
	S	Count	6	3	9	
		% of total	10.0%	5.0%	15.0%	
Total		Count	36	24	60	
		% of total	60.0%	40.0%	100.0%	
Aminoglycosides (Tobramycin)	I	Count	0	0	0	0.767837
		% of total	0%	0%	0%	
	R	Count	31	20	51	
		% of total	51.7%	33.3%	85.0%	
	S	Count	5	4	9	
		% of total	8.3%	6.7%	15.0%	
Total		Count	36	24	60	
		% of total	60.0%	40.0%	100.0%	
Cephalosporins (Cefotaxime)	I	Count	0	0	0	0.216801
		% of total	0%	0%	0%	
	R	Count	36	23	59	
		% of total	60.0%	38.3%	98.3%	
	S	Count	0	1	1	
		% of total	0.0%	1.7%	1.7%	
Total		Count	36	24	60	
		% of total	60.0%	40.0%	100.0%	
Cephalosporins (Ceftriaxone)	I	Count	0	0	0	0.216801
		% of total	0%	0%	0%	
	R	Count	36	23	59	
		% of total	60.0%	38.3%	98.3%	
	S	Count	0	1	1	
		% of total	0.0%	1.7%	1.7%	
Total		Count	36	24	60	
		% of total	60.0%	40.0%	100.0%	
Cephalosporins (Ceftazidime)	I	Count	0	0	0	No statistics computed
		% of total	0%	0%	0%	
	R	Count	36	24	60	
		% of total	60.0%	40.0%	100.0%	
	S	Count	0	0	0	
		% of total	0%	0%	0%	
Total		Count	36	24	60	
		% of total	60.0%	40.0%	100.0%	
Cephalosporins (Cefepime)	I	Count	0	0	0	0.769056
		% of total	0%	0%	0%	
	R	Count	35	23	58	
		% of total	58.3%	38.3%	96.7%	
	S	Count	1	1	2	
		% of total	1.7%	1.7%	3.3%	
Total		Count	36	24	60	
		% of total	60.0%	40.0%	100.0%	
Carbapenems (Meropenem)	I	Count	0	0	0	0.769056
		% of total	0%	0%	0%	
	R	Count	35	23	58	
		% of total	58.3%	38.3%	96.7%	
	S	Count	1	1	2	
		% of total	1.7%	1.7%	3.3%	
Total		Count	36	24	60	
		% of total	60.0%	40.0%	100.0%	
Penicillins (Ampicillin-sulbactam)	I	Count	0	0	0	No statistics computed
		% of total	0%	0%	0%	
	R	Count	36	24	60	
		% of total	60.0%	40.0%	100.0%	
	S	Count	0	0	0	
		% of total	0%	0%	0%	
Total		Count	36	24	60	
		% of total	60.0%	40.0%	100.0%	
Penicillins (Piperacillin)	I	Count	0	0	0	0.216801
		% of total	0%	0%	0%	
	R	Count	36	23	59	
		% of total	60.0%	38.3%	98.3%	
	S	Count	0	1	1	
		% of total	0.0%	1.7%	1.7%	
Total		Count	36	24	60	
		% of total	60.0%	40.0%	100.0%	
Penicillins (Ticarcillin-clavulanate)	I	Count	0	0	0	0.216801
		% of total	0%	0%	0%	
	R	Count	36	23	59	
		% of total	60.0%	38.3%	98.3%	
	S	Count	0	1	1	
		% of total	0.0%	1.7%	1.7%	
Total		Count	36	24	60	
		% of total	60.0%	40.0%	100.0%	
Fluoroquinolones (Ciprofloxacin)	I	Count	0	0	0	0.769056
		% of total	0%	0%	0%	
	R	Count	35	23	58	
		% of total	58.3%	38.3%	96.7%	
	S	Count	1	1	2	
		% of total	1.7%	1.7%	3.3%	
Total		Count	36	24	60	
		% of total	60.0%	40.0%	100.0%	
Sulfonamide (Trimethoprim/sulfamethoxazole)	I	Count	0	0	0	0.672604
		% of total	0%	0%	0%	
	R	Count	34	22	56	
		% of total	56.7%	36.7%	93.3%	
	S	Count	2	2	4	
		% of total	3.3%	3.3%	6.7%	
Total		Count	36	24	60	
		% of total	60.0%	40.0%	100.0%	
Tetracyclines (Minocycline)	I	Count	0	0	0	0.216801
		% of total	0%	0%	0%	
	R	Count	36	23	59	
		% of total	60.0%	38.3%	98.3%	
	S	Count	0	1	1	
		% of total	0.0%	1.7%	1.7%	
Total		Count	36	24	60	
		% of total	60.0%	40.0%	100.0%	
Polymyxins (Colistin)	I	Count	0	0	0	No statistics computed
		% of total	0%	0%	0%	
	R	Count	36	24	60	
		% of total	60.0%	40.0%	100.0%	
	S	Count	0	0	0	
		% of total	0%	0%	0%	
Total		Count	36	24	60	
		% of total	60.0%	40.0%	100.0%	
***relBE***						
Aminoglycosides (Gentamicin)	I	Count	0	0	0	0.178467
		% of total	0%	0%	0%	
	R	Count	28	30	58	
		% of total	46.7%	50.0%	96.7%	
	S	Count	0	2	2	
		% of total	0.0%	3.3%	3.3%	
Total		Count	28	32	60	
		% of total	46.7%	53.3%	100.0%	
Aminoglycosides (Amikacin)	I	Count	0	0	0	0.884756
		% of total	0%	0%	0%	
	R	Count	24	27	51	
		% of total	40.0%	45.0%	85.0%	
	S	Count	4	5	9	
		% of total	6.7%	8.3%	15.0%	
Total		Count	28	32	60	
		% of total	46.7%	53.3%	100.0%	
Aminoglycosides (Tobramycin)	I	Count	0	0	0	0.384488
		% of total	0%	0%	0%	
	R	Count	25	26	51	
		% of total	41.7%	43.3%	85.0%	
	S	Count	3	6	9	
		% of total	5.0%	10.0%	15.0%	
Total		Count	28	32	60	
		% of total	46.7%	53.3%	100.0%	
Cephalosporins (Cefotaxime)	I	Count	0	0	0	0.281004
		% of total	0%	0%	0%	
	R	Count	27	32	59	
		% of total	45.0%	53.3%	98.3%	
	S	Count	1	0	1	
		% of total	1.7%	0.0%	1.7%	
Total		Count	28	32	60	
		% of total	46.7%	53.3%	100.0%	
Cephalosporins (Ceftriaxone)	I	Count	0	0	0	0.345523
		% of total	0%	0%	0%	
	R	Count	28	31	59	
		% of total	46.7%	51.7%	98.3%	
	S	Count	0	1	1	
		% of total	0.0%	1.7%	1.7%	
Total		Count	28	32	60	
		% of total	46.7%	53.3%	100.0%	
Cephalosporins (Ceftazidime)	I	Count	0	0	0	No statistics computed
		% of total	0%	0%	0%	
	R	Count	28	32	60	
		% of total	46.7%	53.3%	100.0%	
	S	Count	0	0	0	
		% of total	0%	0%	0%	
Total		Count	28	32	60	
		% of total	46.7%	53.3%	100.0%	
Cephalosporins (Cefepime)	I	Count	0	0	0	0.178467
		% of total	0%	0%	0%	
	R	Count	28	30	58	
		% of total	46.7%	50.0%	96.7%	
	S	Count	0	2	2	
		% of total	0.0%	3.3%	3.3%	
Total		Count	28	32	60	
		% of total	46.7%	53.3%	100.0%	
Carbapenems (Meropenem)	I	Count	0	0	0	0.923436
		% of total	0%	0%	0%	
	R	Count	27	31	58	
		% of total	45.0%	51.7%	96.7%	
	S	Count	1	1	2	
		% of total	1.7%	1.7%	3.3%	
Total		Count	28	32	60	
		% of total	46.7%	53.3%	100.0%	
Penicillins (Ampicillin-sulbactam)	I	Count	0	0	0	No statistics computed
		% of total	0%	0%	0%	
	R	Count	28	32	60	
		% of total	46.7%	53.3%	100.0%	
	S	Count	0	0	0	
		% of total	0%	0%	0%	
Total		Count	28	32	60	
		% of total	46.7%	53.3%	100.0%	
Penicillins (Piperacillin)	I	Count	0	0	0	0.345523
		% of total	0%	0%	0%	
	R	Count	28	31	59	
		% of total	46.7%	51.7%	98.3%	
	S	Count	0	1	1	
		% of total	0.0%	1.7%	1.7%	
Total		Count	28	32	60	
		% of total	46.7%	53.3%	100.0%	
Penicillins (Ticarcillin-clavulanate)	I	Count	0	0	0	0.281004
		% of total	0%	0%	0%	
	R	Count	27	32	59	
		% of total	45.0%	53.3%	98.3%	
	S	Count	1	0	1	
		% of total	1.7%	0.0%	1.7%	
Total		Count	28	32	60	
		% of total	46.7%	53.3%	100.0%	
Fluoroquinolones (Ciprofloxacin)	I	Count	0	0	0	0.178467
		% of total	0%	0%	0%	
	R	Count	28	30	58	
		% of total	46.7%	50.0%	96.7%	
	S	Count	0	2	2	
		% of total	0.0%	3.3%	3.3%	
Total		Count	28	32	60	
		% of total	46.7%	53.3%	100.0%	
Sulfonamide (Trimethoprim/sulfamethoxazole)	I	Count	0	0	0	0.239704
		% of total	0%	0%	0%	
	R	Count	25	31	56	
		% of total	41.7%	51.7%	93.3%	
	S	Count	3	1	4	
		% of total	5.0%	1.7%	6.7%	
Total		Count	28	32	60	
		% of total	46.7%	53.3%	100.0%	
Tetracyclines (Minocycline)	I	Count	0	0	0	0.345523
		% of total	0%	0%	0%	
	R	Count	28	31	59	
		% of total	46.7%	51.7%	98.3%	
	S	Count	0	1	1	
		% of total	0.0%	1.7%	1.7%	
Total		Count	28	32	60	
		% of total	46.7%	53.3%	100.0%	
Polymyxins (Colistin)	I	Count	0	0	0	No statistics computed
		% of total	0%	0%	0%	
	R	Count	28	32	60	
		% of total	46.7%	53.3%	100.0%	
	S	Count	0	0	0	
		% of total	0%	0%	0%	
Total		Count	28	32	60	
		% of total	46.7%	53.3%	100.0%	
***higBA***						
Aminoglycosides (Gentamicin)	I	Count	0	0	0	0.224114
		% of total	0%	0%	0%	
	R	Count	25	33	58	
		% of total	41.7%	55.0%	96.7%	
	S	Count	0	2	2	
		% of total	0.0%	3.3%	3.3%	
Total		Count	25	35	60	
		% of total	41.7%	58.3%	100.0%	
Aminoglycosides (Amikacin)	I	Count	0	0	0	0.199360
		% of total	0%	0%	0%	
	R	Count	23	28	51	
		% of total	38.3%	46.7%	85.0%	
	S	Count	2	7	9	
		% of total	3.3%	11.7%	15.0%	
Total		Count	25	35	60	
		% of total	41.7%	58.3%	100.0%	
Aminoglycosides (Tobramycin)	I	Count	0	0	0	0.199360
		% of total	0%	0%	0%	
	R	Count	23	28	51	
		% of total	38.3%	46.7%	85.0%	
	S	Count	2	7	9	
		% of total	3.3%	11.7%	15.0%	
Total		Count	25	35	60	
		% of total	41.7%	58.3%	100.0%	
Cephalosporins (Cefotaxime)	I	Count	0	0	0	0.394055
		% of total	0%	0%	0%	
	R	Count	25	34	59	
		% of total	41.7%	56.7%	98.3%	
	S	Count	0	1	1	
		% of total	0.0%	1.7%	1.7%	
Total		Count	25	35	60	
		% of total	41.7%	58.3%	100.0%	
Cephalosporins (Ceftriaxone)	I	Count	0	0	0	0.394055
		% of total	0%	0%	0%	
	R	Count	25	34	59	
		% of total	41.7%	56.7%	98.3%	
	S	Count	0	1	1	
		% of total	0.0%	1.7%	1.7%	
Total		Count	25	35	60	
		% of total	41.7%	58.3%	100.0%	
Cephalosporins (Ceftazidime)	I	Count	0	0	0	No statistics computed
		% of total	0%	0%	0%	
	R	Count	25	35	60	
		% of total	41.7%	58.3%	100.0%	
	S	Count	0	0	0	
		% of total	0%	0%	0%	
Total		Count	25	35	60	
		% of total	41.7%	58.3%	100.0%	
Cephalosporins (Cefepime)	I	Count	0	0	0	0.224114
		% of total	0%	0%	0%	
	R	Count	25	33	58	
		% of total	41.7%	55.0%	96.7%	
	S	Count	0	2	2	
		% of total	0.0%	3.3%	3.3%	
Total		Count	25	35	60	
		% of total	41.7%	58.3%	100.0%	
Carbapenems (Meropenem)	I	Count	0	0	0	0.224114
		% of total	0%	0%	0%	
	R	Count	25	33	58	
		% of total	41.7%	55.0%	96.7%	
	S	Count	0	2	2	
		% of total	0.0%	3.3%	3.3%	
Total		Count	25	35	60	
		% of total	41.7%	58.3%	100.0%	
Penicillins (Ampicillin-sulbactam)	I	Count	0	0	0	No statistics computed
		% of total	0%	0%	0%	
	R	Count	25	35	60	
		% of total	41.7%	58.3%	100.0%	
	S	Count	0	0	0	
		% of total	0%	0%	0%	
Total		Count	25	35	60	
		% of total	41.7%	58.3%	100.0%	
Penicillins (Piperacillin)	I	Count	0	0	0	0.394055
		% of total	0%	0%	0%	
	R	Count	25	34	59	
		% of total	41.7%	56.7%	98.3%	
	S	Count	0	1	1	
		% of total	0.0%	1.7%	1.7%	
Total		Count	25	35	60	
		% of total	41.7%	58.3%	100.0%	
Penicillins (Ticarcillin-clavulanate)	I	Count	0	0	0	0.394055
		% of total	0%	0%	0%	
	R	Count	25	34	59	
		% of total	41.7%	56.7%	98.3%	
	S	Count	0	1	1	
		% of total	0.0%	1.7%	1.7%	
Total		Count	25	35	60	
		% of total	41.7%	58.3%	100.0%	
Fluoroquinolones (Ciprofloxacin)	I	Count	0	0	0	0.224114
		% of total	0%	0%	0%	
	R	Count	25	33	58	
		% of total	41.7%	55.0%	96.7%	
	S	Count	0	2	2	
		% of total	0.0%	3.3%	3.3%	
Total		Count	25	35	60	
		% of total	41.7%	58.3%	100.0%	
Sulfonamide (Trimethoprim/sulfamethoxazole)	I	Count	0	0	0	0.726393
		% of total	0%	0%	0%	
	R	Count	23	33	56	
		% of total	38.3%	55.0%	93.3%	
	S	Count	2	2	4	
		% of total	3.3%	3.3%	6.7%	
Total		Count	25	35	60	
		% of total	41.7%	58.3%	100.0%	
Tetracyclines (Minocycline)	I	Count	0	0	0	0.394055
		% of total	0%	0%	0%	
	R	Count	25	34	59	
		% of total	41.7%	56.7%	98.3%	
	S	Count	0	1	1	
		% of total	0.0%	1.7%	1.7%	
Total		Count	25	35	60	
		% of total	41.7%	58.3%	100.0%	
Polymyxins (Colistin)	I	Count	0	0	0	No statistics computed
		% of total	0%	0%	0%	
	R	Count	25	35	60	
		% of total	41.7%	58.3%	100.0%	
	S	Count	0	0	0	
		% of total	0%	0%	0%	
Total		Count	25	35	60	
		% of total	41.7%	58.3%	100.0%	

**Table 6:** The mazEF, *relBE*, and *higBA* prevalence of the clinical isolates and their relationship with antibiotic resistance status

Investigated gene			Negative	Positive	Total	*P* value
*mazEF*	MDR	Count	5	5	10	0.535261
		% of total	8.3%	8.3%	16.7%	
	PDR	Count	27	18	45	
		% of total	45.0%	30.0%	75.0%	
	XDR	Count	4	1	5	
		% of total	6.7%	1.7%	8.3%	
Total		Count	36	24	60	
		% of total	60.0%	40.0%	100.0%	
*relBE*	MDR	Count	5	5	10	0.782285
		% of total	8.3%	8.3%	16.7%	
	PDR	Count	20	25	45	
		% of total	33.3%	41.7%	75.0%	
	XDR	Count	3	2	5	
		% of total	5.0%	3.3%	8.3%	
Total		Count	28	32	60	
		% of total	46.7%	53.3%	100.0%	
*higBA*	MDR	Count	3	7	10	0.701674
		% of total	5.0%	11.7%	16.7%	
	PDR	Count	20	25	45	
		% of total	33.3%	41.7%	75.0%	
	XDR	Count	2	3	5	
		% of total	3.3%	5.0%	8.3%	
Total		Count	25	35	60	
		% of total	41.7%	58.3%	100.0%	

It was also discovered that the sex of the patients from whom the *A. baumannii* isolates were collected had no significant effects on the presence or the absence of the *mazEF*, *relBE*, and *higBA* genes in the bacterial isolates (*p* = 0.747, *p* = 0.139, and *p* = 0.109, respectively). In reference to the age of the patients from whom the clinical isolates were collected and the presence or the absence of the *mazEF*, *relBE*, and *higBA* genes, no significant relationship was found according to the results (*p* = 0.555, *p* = 0.894, and *p* = 0.390, respectively). Additionally, the sources from which the *A. baumannii* isolates were derived had no significant effect on the presence or the absence of the *mazEF*, *relBE*, and *higBA* genes in the bacterial isolates (*p* = 0.198, *p* = 0.437, and *p* = 0.741, respectively). Moreover, it was also revealed that there is no significant relationship between the patients’ hospitalization ward and the presence or the absence of the *mazEF*, *relBE*, and *higBA* genes in the clinical isolates (*p* = 0.504, *p* = 0.615, and *p* = 0.384, respectively). Ultimately, it was elucidated that the cause of hospitalization had no significant correlation with the presence or the absence of the *mazEF*, *relBE*, and *higBA* genes (*p* = 0.487, *p* = 0.552, and *p* = 0.381, respectively).

### Nanoparticle characterization

DLS results demonstrated that the CS nanoparticles had a mean size of 180.1 ± 7.2 nm with a PI of 0.35 while the MP-CS nanoparticles demonstrated an average size of 191.5 ± 5.3 nm with a PI of 0.32. Moreover, the zeta potential of the CS and MP-CS nanoparticles were 24.8 and 27.3 mV, respectively.

#### Encapsulation efficiency

**Fig. 1:**
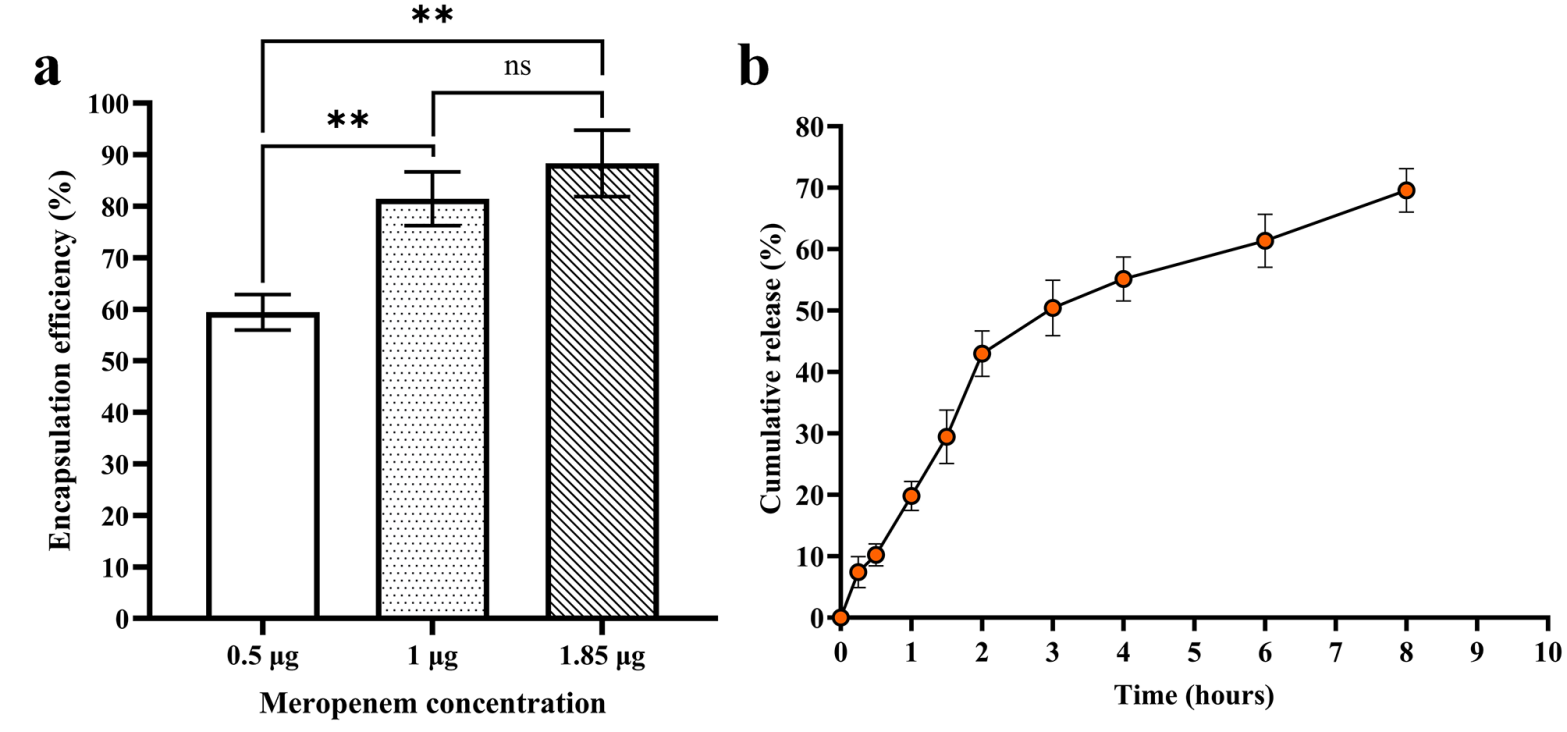
The encapsulation efficiency (**a**) and drug release test (**b**) of the MP-CS nanoparticles. Data are expressed as mean ± standard deviation. All experiments were performed in triplicate (*n* = 3). ** and *ns* represent *p* < 0.01 and *p* > 0.05, respectively

Herein, we investigated the encapsulation efficiency of the prepared nanoparticles using three different concentrations of meropenem (0.5, 1, and 1.85 µg). According to our results (Fig. 1a), the nanoparticles demonstrated an encapsulation efficiency of 59.42 ± 3.43% at the concentration of 0.5 µg, which was significantly lower than the concentrations of 1 and 1.85 µg with the encapsulation efficiencies of 81.44 ± 5.23 and 88.32 ± 6.46%, respectively (*p* < 0.01 for both comparisons). Of note, the concentrations of 1 and 1.85 µg meropenem did not show any significant difference in their encapsulation efficiency rates. Therefore, the concentration of 1.85 µg was selected for the rest of the experiment.

#### In vitro drug release

Our meropenem-loaded nanoparticles demonstrated an in vitro release rate of around 69% after 8 h (Fig. 1b). A burst release pattern was observed during the first two hours as the nanoparticles had release rates of 7.435 ± 2.534, 10.243 ± 1.795, 19.812 ± 2.353, 29.44 ± 4.364, and 43 ± 3.694% at 15 min, 30 min, 60 min, 90 min, and 2 h, respectively. After this, the release pattern of the nanoparticles shifted toward a more gradual behavior, exhibiting release rates of 50.439 ± 4.543, 55.145 ± 3.598, 61.345 ± 4.342, and 69.574 ± 3.534% at 3 h, 4 h, 6 h, and 8 h, respectively.

### Antibacterial effects of the nanoparticles

**Fig. 2:**
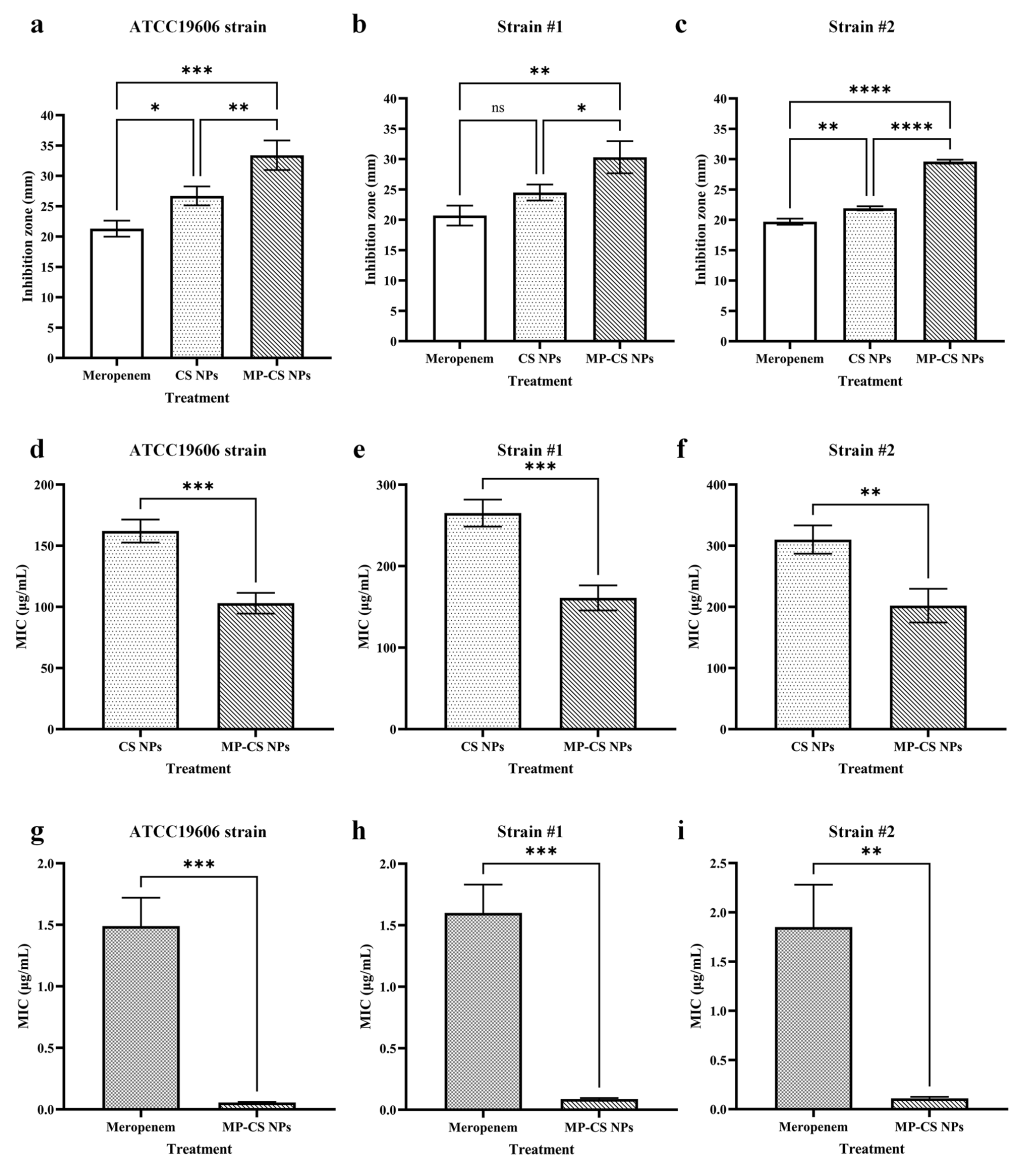
Assessment of the antibacterial effects of the MP-CS nanoparticles. The antibacterial effects of meropenem, the CS nanoparticles, and the MP-CS nanoparticles on *A. baumannii* (ATCC19606) strain (**a**), *A. baumannii* strain #1 (**b**), and *A. baumannii* strain #2 (**c**) assessed via the well diffusion method. The minimum inhibitory concentrations of the CS nanoparticles and MP-CS nanoparticles on *A. baumannii* (ATCC19606) strain (**d**), *A. baumannii* strain #1 (**e**), and *A. baumannii* strain #2 (**f**). The minimum inhibitory concentrations of meropenem and meropenem encapsulated in the MP-CS nanoparticles (at their corresponding minimum inhibitory concentrations) on *A. baumannii* (ATCC19606) strain (**g**), *A. baumannii* strain #1 (**h**), and *A. baumannii* strain #2 (**i**). NPs, nanoparticles. Data are expressed as mean ± standard deviation. All experiments were performed in triplicate (*n* = 3). *, **, ***, ****, and *ns* represent *p* < 0.05, *p* < 0.01, *p* < 0.001, *p* < 0.0001, and *p* > 0.05, respectively

In regard to the in vitro antibacterial activity of the nanoparticles, a well diffusion assay was conducted. In regard to the *A. baumannii* (ATCC19606) strain (Fig. 2a), meropenem, the CS nanoparticles, and the MP-CS nanoparticles demonstrated the lowest to highest inhibition zones, respectively. Moreover, the inhibition zones caused by the loaded and unloaded nanoparticles were both significantly higher than those of free meropenem (*p* < 0.05 and *p* < 0.001, respectively). Furthermore, the MP-CS nanoparticles caused inhibition zones significantly greater than those of the CS nanoparticles (*p* < 0.01). In regard to clinical isolate #1 (Fig. 2b), meropenem, the CS nanoparticles, and the MP-CS nanoparticles demonstrated the lowest to highest inhibition zones, respectively. However, the CS nanoparticles did not cause inhibition zones significantly different from those caused by free meropenem. On the other hand, the inhibition zone caused by the MP-CS nanoparticles was significantly greater than those of the free meropenem and the CS nanoparticles (*p* < 0.01 and *p* < 0.05, respectively). In regard to clinical isolate #2 (Fig. 2c), the greatest inhibition zone was caused by the MP-CS nanoparticles followed by the CS nanoparticles and free meropenem. The antibacterial effects of the MP-CS nanoparticles were significantly higher than those caused by free meropenem and the CS nanoparticles (*p* < 0.0001 for both comparisons). Moreover, the CS nanoparticles also caused inhibition zones significantly greater than those mediated by free meropenem (*p* < 0.01).

In regard to MIC, the MP-CS nanoparticles demonstrated significantly lower MIC values against the *A. baumannii* (ATCC19606) strain, *A. baumannii* strain #1, and *A. baumannii* strain #2 in comparison with their unloaded counterparts (103 vs. 162, 161 vs. 265, and 202 vs. 310 µg/mL, respectively; *p* < 0.001, *p* < 0.001, *p* < 0.01, respectively) (Fig. 2d, e, and **f**, respectively). A comparison between the concentrations of the meropenem encapsulated in the MP-CS nanoparticles (calculated at the corresponding MIC concentrations of the MP-CS nanoparticles) with the concentration of free meropenem demonstrated that a significantly lower concentration of the antibiotic was required to reach the MIC value against the *A. baumannii* (ATCC19606) strain, *A. baumannii* strain #1, and *A. baumannii* strain #2 (0.0558 vs. 1.49, 0.0873 vs. 1.6, and 0.1096 vs. 1.85 µg/mL, respectively; *p* < 0.001, *p* < 0.001, *p* < 0.01, respectively) (Fig. 2g, h, and **i**, respectively).

### CFU counting

The results of the CFU counting experiment, at different stages of *A. baumannii* infection in the presence and absence of the MP-CS nanoparticles have been summarized in Table 7. The CFU count of the bacteria internalized into A549 and NCI-H292 cells was around 14–26% when the cells were co-cultured with the *A. baumannii* strains. However, this count remarkably dropped to around 1–4% when the cells were treated with MP-CS nanoparticles during the co-infection step. Moreover, the CFU count of bacteria interacting or adhering on the surface of A549 or NCI-H292 cells dropped from around 30–37% to 2-3.5% while the cells were under the MP-CS nanoparticle treatment. Moreover, the CFU count of bacteria remaining non-interacting during coinfection was around 93–96% when the cells were treated with the MP-CS nanoparticles during the co-infection step.

**Table 7:** The colony-forming unit (CFU) counting of different *A. baumannii* isolates at different stages of infection in the absence and presence of the MP-Cs nanoparticles

Tested strain + Treatment	Bacteria used for coinfection	Bacteria remaining non-interacting during coinfection	Bacteria interacting or adhered on the surface of A549 cells	Bacteria internalized into A549 cells
A549 cells				
A549 + A. baumannii ATCC	12 × 10^6^ (100%)	5.57 × 10^6^ (46.49%)	3.89 × 10^6^ (32.43%)	2.52 × 10^6^ (21.08%)
A549 + A. baumannii ATCC + MP-CS nanoparticles	12 × 10^6^ (100%)	11.53 × 10^6^ (96.16%)	3.18 × 10^5^ (2.65%)	1.42 × 10^5^ (1.19%)
A549 + A. baumannii Strain #1	12 × 10^6^ (100%)	5.22 × 10^6^ (43.51%)	3.81 × 10^6^ (31.77%)	2.96 × 10^6^ (24.72%)
A549 + A. baumannii Strain #1 + MP-CS nanoparticles	12 × 10^6^ (100%)	11.43 × 10^6^ (95.25%)	3.52 × 10^5^ (2.94%)	2.17 × 10^5^ (1.81%)
A549 + A. baumannii Strain #2	12 × 10^6^ (100%)	4.81 × 10^6^ (40.13%)	4.14 × 10^6^ (34.50%)	3.04 × 10^6^ (25.37%)
A549 + A. baumannii Strain #2 + MP-CS nanoparticles	12 × 10^6^ (100%)	11.36 × 10^6^ (94.73%)	3.73 × 10^5^ (3.11%)	2.59 × 10^5^ (2.16%)
NCI-H292 cells				
NCI-H292 + A. baumannii ATCC	12 × 10^6^ (100%)	4.81 × 10^6^ (40.14%)	4.46 × 10^6^ (37.19%)	2.72 × 10^6^ (22.67%)
NCI-H292 + A. baumannii ATCC + MP-CS nanoparticles	12 × 10^6^ (100%)	11.36 × 10^6^ (94.67%)	3.49 × 10^5^ (2.91%)	2.90 × 10^5^ (2.42%)
NCI-H292 + A. baumannii Strain #1	12 × 10^6^ (100%)	5.26 × 10^6^ (43.89%)	3.65 × 10^6^ (30.45%)	3.07 × 10^6^ (25.66%)
NCI-H292 + A. baumannii Strain #1 + MP-CS nanoparticles	12 × 10^6^ (100%)	11.22 × 10^6^ (93.58%)	4.2 × 10^5^ (3.5%)	3.5 × 10^5^ (2.92%)
NCI-H292 + A. baumannii Strain #2	12 × 10^6^ (100%)	5.79 × 10^6^ (48.32%)	4.50 × 10^6^ (37.55%)	1.69 × 10^6^ (14.13%)
NCI-H292 + A. baumannii Strain #2 + MP-CS nanoparticles	12 × 10^6^ (100%)	11.29 × 10^6^ (94.12%)	2.56 × 10^5^ (2.14%)	4.48 × 10^5^ (3.74%)

### Cytotoxic effects of the nanoparticles on A549 and NCI-H292 cells

**Fig. 3:**
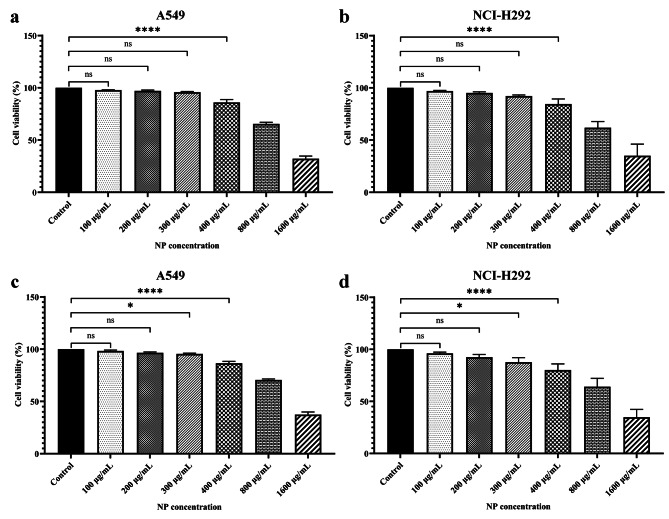
Assessment of the effect of MP-CS nanoparticles on the viability of A549 and NCI-H292 cells via the MTT (**a** and **b, respectively**) and LDH assays (**c** and **d, respectively**). Data are expressed as mean ± standard deviation. NPs, nanoparticles. All experiments were performed in triplicate (*n* = 3). *, ****, and *ns* represent *p* < 0.05, *p* < 0.0001, and *p* > 0.05, respectively

The cytotoxic effects of the MP-CS nanoparticles on the viability of A549 and NCI-H292 cells were assessed via the MTT and LDH assays. The results of the MTT assay demonstrated that the concentrations at which the nanoparticles exhibited antibacterial activity had no negative effect on the viability of A549 and NCI-H292 cells (Fig. 3a and **b**). Of note, the IC50 values for the MP-CS nanoparticles were calculated as 726.9 µg/mL (R^2^ = 0.9748) against the A549 cells and 643.1 µg/mL (R^2^ = 0.9709) against the NCI-H292 cells, which were around four-fold higher than the concentration at which the nanoparticles showed antibacterial activity (meaning MIC). In regard to the results of the MTT assay, the concentrations of 100, 200, and 300 µg/mL of the MP-CS nanoparticle did not significantly reduce the viability of A549 and NCI-H292 cells in comparison with those of the control groups. However, at the concentration of 400 µg/mL, the cell viability rates of both A549 and NCI-H292 cells declined to around 86.04 and 84.5%, respectively, which were significantly lower in comparison with those of the corresponding control groups (*p* < 0.0001).

In contrast with the MTT assay results, which determine the number of living cells, the LDH assay measures the number of dead cells. Briefly, LDH is released into the cell culture medium upon cell membrane disruption and cell death which could be correlated to cell death rate. For the simplification of the comparison between the results of the MTT assay and LDH assay, we expressed the results of the LDH assay as the percentage of the living cells. According to our findings (Fig. 3c and **d**), the MP-CS nanoparticles at the concentrations of 100 and 200 µg/mL did not significantly reduce the viability of A549 and NCI-H292 cells in comparison with those of the corresponding control groups. However, a significant reduction in the viability of A549 and NCI-H292 cells was observed at the concentrations of 300 and 400 µg/mL in comparison with those of the corresponding control groups (*p* < 0.05 and *p* < 0.0001, respectively).

### Evaluation of ROS formation

**Fig. 4:**
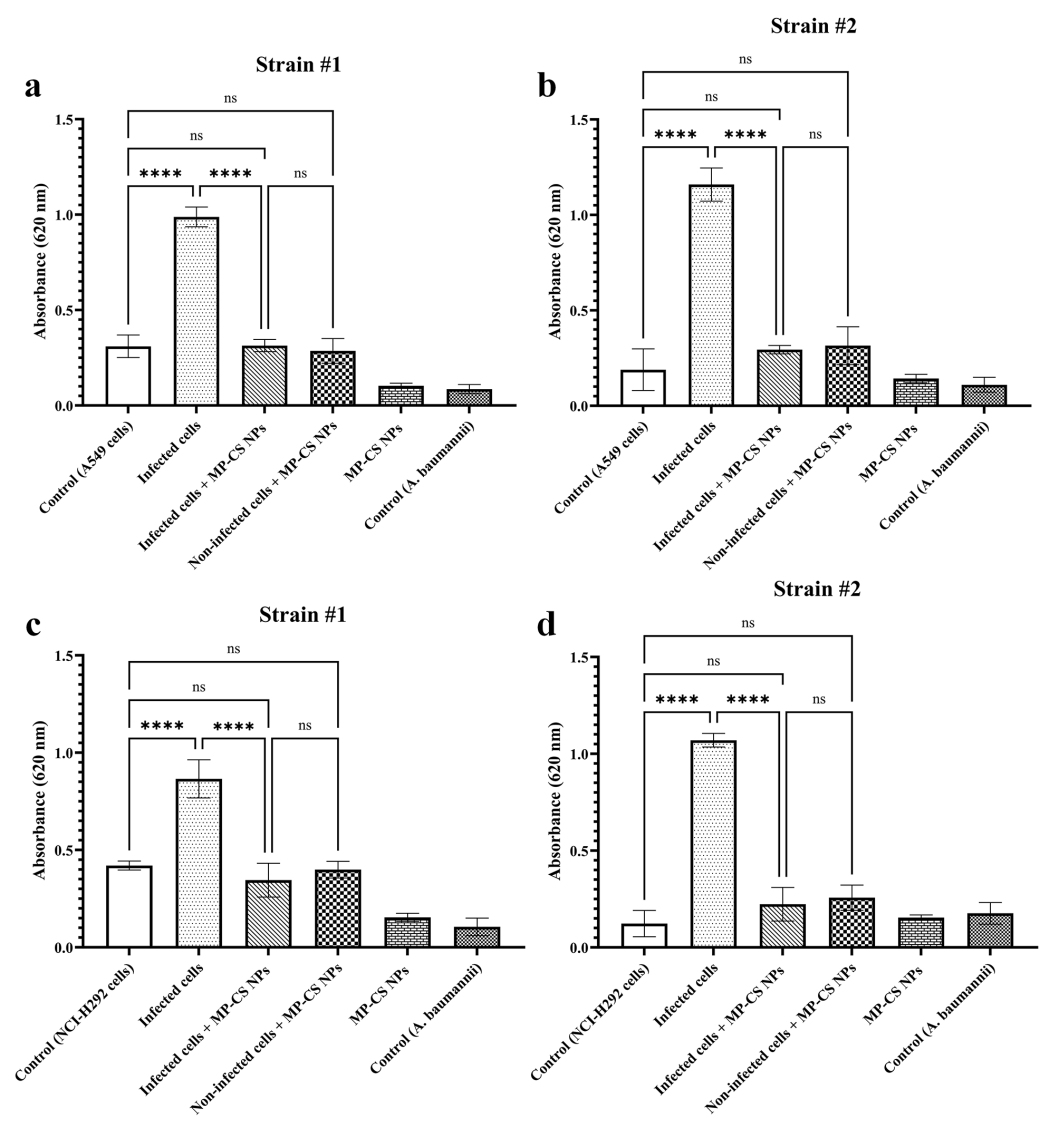
ROS level evaluation test. **a** and **b**: A549 cells. **c** and **d**: NCI-H292 cells. Data are expressed as mean ± standard deviation. NPs, nanoparticles. All experiments were performed in triplicate (*n* = 3). **** and *ns* represent *p* < 0.0001 and *p* > 0.05, respectively

Our results demonstrated that the infection of A549 and NCI-H292 cells with *A. baumannii* significantly increased the level of formed ROS in comparison with those of the uninfected cell groups (*p* < 0.0001 for both cell lines) (Fig. 4). However, treatment of the infected cells with the MP-CS nanoparticles significantly lowered the ROS level in comparison with those of the corresponding infected cells (*p* < 0.0001 for both cell lines). Moreover, it was also demonstrated that the levels of ROS following the treatment of the infected A549 and NCI-H292 cells with the MP-CS nanoparticles were comparable to those of the uninfected (control) corresponding cells (*p* > 0.05 for both comparisons), demonstrating no negative effects on the viability of the infected cells as a result of the MP-CS nanoparticle treatment.

## Discussion

In this study, out of 240 clinical specimens, 60 (25%) isolates were identified as *A. baumannii*. Such infection frequency was consistent with that of a study by Babapour and colleagues in which 24% of the bacterial isolates collected from patients in three hospitals in Tehran, Iran were identified as *A. baumannii* [39]. A comparison between the results of this study with those of other similar studies in other countries demonstrates that the infection frequency of this bacterium in clinical isolates collected in Iran is higher than those of the other countries [40, 41]. The reason for this higher *A. baumannii* infection rate in Iranian hospitalized patients can be a subject of further investigation.

According to our results, the highest rate of *A. baumannii* isolates was collected from the trachea specimens (31 isolates; 51.7%), which is consistent with the findings reported in a study by Alkasaby and colleagues [42]. Furthermore, as reported by Maghraby et al., the majority of *A. baumannii* isolates were related to chest infection specimens [26]. However, Al Mobarak et al. reported some contradicting results in this regard as they reported that *A. baumannii* isolates were mainly isolated from wounds, respiratory secretions, urine, and blood [43]. Such differences can be elucidated and justified by studying a larger number of specimens, the underlying medical conditions that led to the hospitalization of the patients, and the general hospitalization conditions.

Based on our results, all of the 60 *A. baumannii* isolates (100%) were resistant to ampicillin, ceftazidime, and colistin; therefore, these antibiotics were designated the most infective ones. On the other hand, as 9 isolates (15%) demonstrated susceptibility to amikacin and tobramycin, these two antibiotics were regarded as the most effective antibiotics in this study. However, since a high rate of resistance to antibiotics by the bacterial isolates was observed in this investigation, these findings are consistent with the findings of previous studies which reported that most *A. baumannii* strains isolated in Iran were resistant to first-line antibiotics, including aminoglycosides, fluoroquinolones, and carbapenems [44–46].

The widespread resistance of *A. baumannii* to different groups of antimicrobial agents has been previously reported by various studies conducted in Iran. For instance, in a study by Fazeli et al. in which 121 isolates of *A. baumannii* were isolated from different hospitals in Isfahan, Iran, the investigators reported that these bacterial strains were highly resistant to common antibiotics including cefotaxime (100%), ceftriaxone (100%), meropenem (100%), ciprofloxacin (100%), aztreonam (100%), cefepime (99.2%), trimethoprim-sulfamethoxazole (99.2%), tetracycline (92.6%), amikacin (87.6%), tobramycin (86.8%), and ampicillin-sulbactam (33.9%) [47]. Moreover, Karimi et al. reported that among 60 *A. baumannii* isolates obtained from *Hazrat-e-Rasoul Hospital* in Tehran, 93.3% were resistant to ceftazidime and 91.6% were resistant to amikacin, which is consistent with our results. Also, they reported that 3.3% of their bacterial isolates were resistant to colistin making it the most effective antibiotic [48].

In the current study, 10 (16.7%), 5 (8.3%), and 45 (75%), of the clinical isolates were MDR, XDR, and PDR, respectively. Our results were rather different from those conducted and reported by other researchers in Iran [44, 49–51]. For instance, in a study by Fazeli et al., it was reported that 62.8% of the *A. baumannii* isolates were considered XDR [47]. It is worth mentioning that as the findings of the present study and similar studies conducted in Iran indicate, antibiotic resistance and the emergence of MDR strains of *A. baumannii* are rapidly increasing and this topic requires meticulous molecular and clinical attention [44, 49–51]. Furthermore, novel prevention and treatment strategies against *A. baumannii* infections are urgently warranted.

The prevalence of the TA system genes in the *A. baumannii* isolates was also investigated in this study. The *mazEF*, *relBE*, and *higBA* genes were present in 24 (40%), 32 (53.33%), and 35 (58.3%) bacterial isolates, respectively. In addition, 6 isolates (10%) were negative for the three investigated genes. To our knowledge, only a few studies have investigated the prevalence and functionality of the TA system genes in bacterial isolates. According to a similar study on 27 *A. baumannii* isolates, 62.9% had the *mazEF* gene, 81.5% had the *relBE* gene, and 29.6% had the *higBA* gene [26]. As reported by Ghafourian et al., the chromosomal DNA of all of the *A. baumannii* isolates (100%) were positive for *mazEF*, and that *relBE* was found in the chromosomal DNA of only 88.2% of the bacterial isolates whereas *higBA* was the least prevalent gene (4.7%) in the bacterial isolates [7]. In contrast, as performing PCR on the plasmid DNA of the bacterial isolates did not result in the amplification of any of the mentioned TA system genes, it was concluded that maintenance of the TA systems is through chromosomal genes in *A. baumannii* [7].

Previous studies have also investigated the TA systems in other bacterial species as well. For instance, Savari et al. found that both the *relBE* and *higBA* genes are present in all of the isolates of *P. aeruginosa* investigated [52]. In another study conducted by Hemati et al., the *relBE* gene was present in all of the *P. aeruginosa* isolates investigated [53]. According to Moritz and colleagues, the TA system genes were also prevalent in vancomycin-resistant *enterococci*, with *mazEF* and *relBE* being present in 100 and 44% of the bacterial isolates, respectively [14].

Regarding the characterization of the nanoparticles, other researchers have also used the same technique for the preparation of CS nanoparticles and have reported results consistent with our findings. For instance, Mahboubi Kancha et al. have reported an average size of around 170–200 nm for CS nanoparticles [54, 55]. The zeta potentials of our nanoparticles are also similar to those reported by Mahboubi Kancha et al. as these researchers have reported a zeta potential of around 25–30 mV for their CS nanoparticles [54, 55].

Other researchers have also reported encapsulation efficiency rates of around 75–80% for CS nanoparticles loaded with different drugs other than meropenem [54, 55]. These values are consistent with the findings of our experiments. CS nanoparticles have also been used for the delivery of other antibiotics such as colistin and imipenem. According to an investigation by Elnaggar and colleagues, entrapment efficiency of around 75% for CS nanoparticles loaded colistin has been reported [56]. In a 2023 report, Mufti and colleagues reported an encapsulation efficiency of around 87% for the encapsulation of imipenem into CS nanoparticles [57]. Such findings accentuate the capacity of CS nanoparticles for the encapsulation of antibiotics.

Similar sustained drug release patterns from CS nanoparticles have been reported by other researchers. According to a recent investigation, a drug release rate of around 60% from CS nanoparticles loaded with a particular snake’s venom has been reported [54], which is slightly lower than our reported release rate. According to another investigation, the release rate of berberine from loaded CS nanoparticles has been reported to be around 77% [55]. Based on the findings of Elnaggar et al., around 27% release of colistin from CS nanoparticles after 2 h has been documented which is similar to our findings (which was 43 ± 3.694) [56].

Researchers investigating nanoparticles for the delivery of antibiotics have also reported that such nanoparticles could mediate antibacterial effects themselves. According to Mufti and colleagues, it was demonstrated that CS nanoparticles loaded with imipenem could mediate antibacterial effects in vitro and that the loading of these nanoparticles enhanced their antibacterial impact against *A. baumannii* [57]. Other types of nanoparticles have also been developed and evaluated for the delivery of antibiotics against antibiotic-resistant bacterial strains. For instance, in a report by Shaaban and colleagues, it was demonstrated imipenem/cilastatin-loaded poly Ɛ-caprolactone nano-formulations improved the antibacterial activity of imipenem against imipenem-resistant clinical isolates of *Klebsiella pneumoniae* and *P. aeruginosa* in comparison with free imipenem or cilastatin [58]. In 2023, Gui and colleagues also reported that pH-responsive imipenem-loaded nanocarriers demonstrated synergistic antibacterial effects against *A. baumannii* [59]. These findings are consistent with our results and highlight the applicability of nanoparticles for the delivery of antibiotics against various bacterial pathogens.

CS nanoparticles’ outstanding qualities such as their biocompatibility and low cytotoxic effects have attracted a lot of attention in the field of biomedical research [60–62]. For instance, Friedman et al. have reported that not only CS nanoparticles do not trigger immunological responses but they also demonstrated anti-inflammatory activity as these nanoparticles prevented the production of inflammatory cytokines induced by *Propionibacterium acnes* in human monocytes and keratinocytes [63]. Because of this property, CS nanoparticles can be used in tissue engineering, targeted drug delivery, and other therapeutic interventions [60, 62, 64]. Herein, we demonstrated that CS nanoparticles can be loaded with meropenem to mediate antibacterial effects at concentrations that do not mediate significant cytotoxic effects against A549 and NCI-H292 cells. Our findings are consistent with other similar studies. For instance, researchers have reported that CS nanoparticles cross-linked with either TPP or hydroxypropyl methylcellulose phthalate (HPMCP) do not cause significant cytotoxicity on the viability of mesenchymal stem cells [55].

## Conclusion

Due to the high antibiotic resistance rate, there is an urgent need for effective surveillance to control *A. baumannii* in Iran. Management of treatment, such as assessing the sensitivity of the bacterial strains to certain antibiotics before the treatment of the patients to find the most suitable antibiotic(s), and patient hospitalization until the resolution of the infection can be considered as possible solutions to prevent further spread of the resistant bacterial strains. Herein, we demonstrated that there is no significant correlation between the presence of the *mazEF*, *relBE*, and *higBA* genes and the resistance of the *A. baumannii* clinical isolates to the investigated antibiotics or their MDR, XDR, or PDR status. Moreover, we reported that CS nanoparticles encapsulated with meropenem can mediate antibacterial effects against meropenem-susceptible clinical isolates of *A. baumannii* derived from patients hospitalized in Iran. Such antibacterial effects were mediated in a synergistic fashion as unloaded CS nanoparticles also demonstrated antibacterial effects; therefore, the encapsulation of meropenem into the CS nanoparticles resulted in potent antibacterial effects with significantly lower concentrations of meropenem. Since meropenem is an antibiotic to which a proportion of antibiotic-resistant *A. baumannii* clinical isolates are susceptible, improving the antibacterial effects of this antibiotic can be leveraged to fight *A. baumannii* infections that are susceptible to meropenem and are resistant to other antibiotics. These nano-delivery systems are hoped and believed to provide new a direction for combating drug-resistant bacterial infections.

## Data Availability

All data generated or analysed during this study are included in this published article.

## Abbreviations

UTI: Urinary tract infections
VAP: Ventilator-associated pneumonia
TA: Toxin-antitoxin
CS: Chitosan
MDR: Multidrug-resistant
TPP: Sodium tripolyphosphate
MP-CS: Meropenem-loaded chitosan/sodium tripolyphosphate nanoparticles
TSI: Triple Sugar Iron
O-F: Oxidative Fermentative
SIM: Sulfide Indole Motility
TSB: Tryptic Soy Broth
DDM: Disk diffusion method
CLSI: Clinical and Laboratory Standards Institute
MIC: Minimum inhibitory concentration
XDR: Extensively drug-resistant
PDR: Pandrug-resistant
DDW: Double-distilled water
TPP: Tripolyphosphate
PDI: Polydispersity index
PBS: Phosphate-buffered saline
IBRC: Iranian Biological Resource Center
ATCC: American Type Culture Collection
DMEM: Dulbecco’s Modified Eagle Medium
FBS: fetal bovine serum
HEPES: 4-(2-hydroxyethyl)piperazine-1-ethane-sulfonic acid
EDTA: ethylenediaminetetraacetic acid
CFU: Colony-forming unit
NBT: Nitroblue tetrazolium
DMSO: Dimethyl sulfoxide
HPMCP: Hydroxypropyl methylcellulose phthalate
